# Cardioprotective actions of nitroxyl donor Angeli's salt are preserved in the diabetic heart and vasculature in the face of nitric oxide resistance

**DOI:** 10.1111/bph.15849

**Published:** 2022-04-26

**Authors:** Anida Velagic, Jasmin Chendi Li, Cheng Xue Qin, Mandy Li, Minh Deo, Sarah A. Marshall, Dovile Anderson, Owen L. Woodman, John D. Horowitz, Barbara K. Kemp‐Harper, Rebecca H. Ritchie

**Affiliations:** ^1^ Baker Heart and Diabetes Institute Melbourne VIC Australia; ^2^ Monash Institute of Pharmaceutical Sciences Monash University Melbourne VIC Australia; ^3^ Biomedicine Discovery Institute, Department of Pharmacology Monash University Melbourne VIC Australia; ^4^ The Ritchie Centre, Department of Obstetrics and Gynaecology, School of Clinical Sciences Monash University VIC Australia; ^5^ Basil Hetzel Institute, Queen Elizabeth Hospital University of Adelaide SA Australia

**Keywords:** cardiovascular disease, diabetes, HNO, nitric oxide resistance, nitroxyl, type 2 diabetes

## Abstract

**Background and Purpose:**

The risk of fatal cardiovascular events is increased in patients with type 2 diabetes mellitus (T2DM). A major contributor to poor prognosis is impaired nitric oxide (NO•) signalling at the level of tissue responsiveness, termed NO• resistance. This study aimed to determine if T2DM promotes NO• resistance in the heart and vasculature and whether tissue responsiveness to nitroxyl (HNO) is affected.

**Experimental Approach:**

At 8 weeks of age, male Sprague–Dawley rats commenced a high‐fat diet. After 2 weeks, the rats received low‐dose streptozotocin (two intraperitoneal injections, 35 mg·kg^−1^, over two consecutive days) and continued on the same diet. Twelve weeks later, isolated hearts were Langendorff‐perfused to assess responses to the NO• donor diethylamine NONOate (DEA/NO) and the HNO donor Angeli's salt. Isolated mesenteric arteries were utilised to measure vascular responsiveness to the NO• donors sodium nitroprusside (SNP) and DEA/NO, and the HNO donor Angeli's salt.

**Key Results:**

Inotropic, lusitropic and coronary vasodilator responses to DEA/NO were impaired in T2DM hearts, whereas responses to Angeli's salt were preserved or enhanced. Vasorelaxation to Angeli's salt was augmented in T2DM mesenteric arteries, which were hyporesponsive to the relaxant effects of SNP and DEA/NO.

**Conclusion and Implications:**

This is the first evidence that inotropic and lusitropic responses are preserved, and NO• resistance in the coronary and mesenteric vasculature is circumvented, by the HNO donor Angeli's salt in T2DM. These findings highlight the cardiovascular therapeutic potential of HNO donors, especially in emergencies such as acute ischaemia or heart failure.

## Graphical abstract

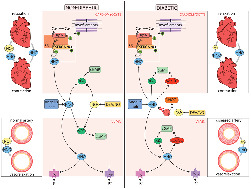

**What is already known**

- Impaired tissue responsiveness to NO•, termed NO• resistance, is documented in diabetic vasculature/platelets.
- NO• resistance contributes to the development of cardiovascular complications in type 2 diabetes.

**What does this study add**

- Type 2 diabetes‐induced NO• resistance in the coronary and mesenteric vasculature is circumvented by nitroxyl.
- Nitroxyl donors act as positive inotropic vasodilators in type 2 diabetes.

**What is the clinical significance**

- Nitroxyl may represent an effective and rapid intervention for cardiovascular emergencies in type 2 diabetes.

**Abbreviations**
HNO: nitroxyl
DEA/NO: diethylamine NONOate
IPA‐NO: iso‐propylamine‐NONOate
STZ: streptozotocin
GTT: glucose tolerance test
ITT: insulin tolerance test
LVSP: left ventricular systolic pressure
LVEDP: left ventricular end‐diastolic pressure
LVDP: left ventricular developed pressure
LV + dP/dt: maximal rate of rise in LV pressure
LV − dP/dt: maximal rate of fall in LV pressure
ODQ: 1*H*‐[1,2,4]oxadiazolo[4,3‐*a*]quinoxaline‐1‐one
KPSS: high K^+^ physiological saline solution
HXC: hydroxocobalamin
LVEF: left ventricular ejection fraction
GSSG: oxidised glutathione
GSH: reduced glutathione
SAM: *S*‐adenosyl‐methionine
γ‐Glu‐Cys: γ‐glutamylcysteine
Cys‐Gly: cysteinylglycine
HFrEF: heart failure with reduced ejection fraction

## 1 INTRODUCTION

Type 2 diabetes mellitus (T2DM) is a growing epidemic with over 463 million individuals affected worldwide, and this number is predicted to increase to 700 million by the year 2045 (International Diabetes Federation, 2019). This rise in prevalence will not only place a greater burden on health care expenditure (Williams et al., 2020) but also will increase the number of patient hospitalisations due to cardiovascular complications, which remain the leading cause of morbidity and mortality in this population (Al‐Salameh et al., 2019). Individuals with T2DM have over a two‐fold increased risk of developing symptomatic heart failure (Ohkuma et al., 2019), a condition for which long‐term prognosis is poor with a 5‐year mortality rate of approximately 75%, irrespective of ejection fraction (Shah et al., 2017). Understanding whether, and if so how, T2DM alters cardiovascular responses to pharmacotherapies employed for the management of cardiovascular pathologies, including in the context of acute heart failure, is essential for improving patient prognosis.

Nitric oxide (NO•) plays an important role in maintaining cardiovascular homeostasis due to its cardio‐ and vaso‐protective effects, which include its ability to inhibit platelet aggregation and inflammation, while promoting vasodilation (Kemp‐Harper & Schmidt, 2009) as well as cardiac relaxation (Paulus & Bronzwaer, 2004). Impaired responsiveness to NO•, termed ‘NO• resistance’, has been identified in the vasculature and platelets of patients with heart failure (Anderson et al., 2004; Maguire et al., 1998) and T2DM (Anderson et al., 2005; Williams et al., 1996). This impairment occurs largely due to elevations in reactive oxygen species (ROS), such as superoxide, which ‘scavenge’ NO• and oxidise its intracellular receptor soluble guanylate cyclase (sGC) (Pacher et al., 2007). Indeed, the degree of impairment in platelet responsiveness to NO• appears to be proportionate to blood glucose levels in patients with diabetes and acute coronary syndromes, which positively correlated with superoxide levels (Worthley et al., 2007). The impact of NO• resistance on long‐term prognosis in patients with diabetes presenting with acute coronary syndromes was highlighted in a study by Schachinger et al., where impaired coronary vasodilation in response to NO• was identified as an independent predictor of adverse cardiovascular events such as myocardial infarction and ischaemic stroke (Schachinger et al., 2000). As such, the therapeutic efficacy of NO• donors in T2DM is likely to be impaired (R.A. Anderson et al., 2005) and thus the need for another therapy that circumvents this problem is paramount.

Nitroxyl (HNO) is the one‐electron reduced product of NO• (Miranda et al., 2003). Similar to its redox sibling, HNO also has anti‐aggregatory (Dautov et al., 2013) and vasodilator (Tare et al., 2017) effects. However, unlike NO•, HNO lacks reactivity with the superoxide anion and thus remains effective during oxidative stress (Leo et al., 2012). Another feature of HNO that distinguishes it from NO• is its ability to induce positive inotropic responses via direct interaction with cysteine residues on thiol‐containing proteins such as ryanodine receptors (Tocchetti et al., 2007) and phospholamban (Keceli et al., 2019) on the sarcoplasmic reticulum Ca^2+^‐ATPase. We have previously demonstrated that inotropic and lusitropic responses to the NO• donor diethylamine‐NONOate (DEA/NO) are impaired, whereas responses to the HNO donor iso‐propylamine‐NONOate (IPA‐NO) are enhanced, in hearts from a rat model of type 1 diabetes mellitus (T1DM) (Qin et al., 2020). However, whether this outcome extends to other HNO donors and the more prevalent subtype of diabetes, that is, T2DM (International Diabetes Federation, 2019), is unclear. Although both subtypes of diabetes are characterised by hyperglycaemia, the pathogenesis and clinical presentation of T2DM is distinct to that of T1DM (Zaccardi et al., 2016). In patients with T2DM, low insulin sensitivity is negatively correlated with left ventricular ejection fraction (LVEF) (Sasso et al., 2000). The main objective of this study was to determine if T2DM promotes NO• resistance in the heart and vasculature, and whether tissue responsiveness to HNO is affected.

## 2 METHODS

### 2.1 Animals and experimental design

All procedures involving animals were approved by the Alfred Research Alliance Animal Ethics Committee (AEC; approval no. E/1759/2017/B) and conducted in accordance with the National Health and Medical Research Council (NMHRC) Australian code for the care and use of animals for scientific purposes and the Guide for the Care and Use of Laboratory Animals published by the United States National Institutes of Health (NIH, 8th edition, revised 2011). Animal studies are reported in compliance with the ARRIVE guidelines (Percie du Sert et al., 2020) and with the recommendations made by the *British Journal of Pharmacology* (Lilley et al., 2020). Group sizes were designed to be equal and account for 15% animal loss in diabetic rats due to failure to develop hyperglycaemia despite streptozotocin (STZ) administration or diabetes‐associated mortality. No formal power calculation was performed regarding the primary outcome of NO• resistance (determined by coronary flow rate to DEA/NO in non‐diabetic vs. diabetic hearts). However, sample sizes were greater than those in our previous work (Qin et al., 2020). In addition, a post hoc power calculation was performed to ensure adequate power using the following online calculator: https://clincalc.com/stats/Power.aspx. Using the mean coronary flow rate response to DEA/NO (non‐diabetic: 7.0 ± 0.7 vs. diabetic: 4.6 ± 0.9), this study had a power of >90% (α = 0.05) to detect a significant difference between two groups with *n* = 8. For all experiments, a flow diagram based on the CONSAERT template is provided for reporting animal use and analysis in preclinical studies (Drucker, 2016), which details animal fate, and any variations/inequalities in sample sizes (Figure S1).

The low‐dose STZ and high‐fat diet rat model is an established model of T2DM, and was utilised for this study as it recapitulates features of disease progression observed in humans (Marsh et al., 2009). Male Sprague–Dawley rats (RRID:RGD_10395233) at three to five  weeks of age were obtained from the Animal Resources Centre (ARC; WA, Australia). Only male rats were used, because female rats are regarded as resistant to the effects of low‐dose STZ (Furman, 2015). All rats were housed in individually ventilated cages (in groups of two to three per cage) and maintained on a 12‐h light/dark cycle at room temperature (22.0 ± 0.1°C) in the PC2‐certified Alfred Research Alliance Precinct Animal Centre. Food (standard laboratory chow) and water were provided ad libitum. Every second day, animal welfare was checked, and paper chip bedding, nesting wool and enrichment devices were replaced. At 6 weeks of age, rats were randomly allocated to non‐diabetic (*n* = 30) or diabetic (*n* = 43) groups using a random number generator. A subset of these animals was allocated to tissue collection only as part of a separate study (detailed in Figure S1). At 8 weeks of age, rats in the diabetic group were placed on a high‐fat diet (SF03‐002; total digestible energy: 59% lipids, 15% protein; wt/wt: 34.6% sucrose; Specialty Feeds, WA, Australia). The difference in colour and consistency between the two diets precluded blinding of experimental groups. Following 2 weeks, the diabetic group received two consecutive daily i.p. injections of STZ (35 mg·kg^−1^ in 0.1‐M citric acid vehicle, pH 4.5; Sigma‐Aldrich, St. Louis, MO, USA), whereas the non‐diabetic group received an equivalent volume of the citric acid vehicle. All animals were maintained on their respective diets for the remainder of the study.

Glucose levels were measured fortnightly, in blood taken from the tail vein and using an Accu‐chek® Performa glucometer (Roche Diagnostics, Basel, Switzerland). When blood glucose levels reached ≥28 mM, rats were administered low‐dose insulin (2 IU every second day, s.c.; Humulin® N intermediate‐acting; Eli Lilly, Indianapolis, IN, USA) to reduce animal welfare burden caused by marked hyperglycaemia. Insulin administration was stopped 1 week prior to the study end‐point. Glucose tolerance tests (GTT) and insulin tolerance tests (ITT) were conducted 1 week prior to end‐point. Rats were fasted for 6 h, after which baseline blood glucose measurement was recorded using a glucometer. At time 0, a single i.p. injection of glucose (d‐glucose, 50% wt/vol, 2 μl·g^−1^; Baxter, Viaflex®) or insulin (0.5 IU·kg^−1^, Humalog® rapid‐acting; Eli Lilly) was administered for the GTT or ITT, respectively. Then, glucose measurements were obtained in tail vein blood at 15, 30, 45, 60, 90, and 120 min. Animal welfare was monitored during and after (2, 4, 24, and 48 h) STZ or citrate vehicle injections, and the GTT or ITT. At 22 weeks of age, rats were anaesthetised using ketamine/xylazine (100/12 mg·kg^−1^, i.p.). Once anaesthetised, whole blood was collected from the portal vein in heparinised tubes, and animals were euthanised via rapid excision of the heart. Whole blood was centrifuged at 1500 rpm for 15 min at 4°C to obtain plasma, which was immediately stored at −80°C for subsequent analysis.

### 2.2 Plasma insulin, triglyceride, and cholesterol levels

Insulin was measured by a rat‐specific ELISA kit, as per the manufacturer's instructions (80‐INSRT‐E01; ALPCO, Salem, NH, USA). Levels of triglycerides (ab65336) and cholesterol (total cholesterol, high‐density lipoprotein [HDL], low‐density lipoprotein [LDL]/very low‐density lipoprotein [VLDL]; ab65390) were quantified according to the manufacturer's instructions (Abcam, Cambridge, UK). Plasma was diluted 1:20 before it was assayed for insulin, triglycerides, and total cholesterol. HDL and LDL/VLDL fractions were diluted 1:4. All dilutions were performed using the appropriate sample buffer specified in the manufacturer's protocol. Natural product studies are reported in compliance with the recommendations made by the *British Journal of Pharmacology* (Izzo et al., 2020).

### 2.3 Perfusion of isolated hearts

Hearts isolated from anaesthetised rats were cannulated via the aorta and Langendorff‐perfused with Krebs' physiological salt solution (PSS, mmol·L^−1^: 118 NaCl, 4.7 KCl, 1.18 MgSO_4_.7H_2_O, 1.12 KH_2_PO_4_, 25 NaHCO_3_, 11 d‐glucose, 0.5 EDTA, and 1.75 CaCl_2_) and continuously bubbled with carbogen (95% O_2_, 5% CO_2_) at 37°C. Coronary flow rate was gradually increased to 10 mL min^−1^. Then, a fluid‐filled latex balloon attached to a pressure transducer was inserted into the left atrium and positioned in the left ventricle to measure left ventricular pressure. Following an equilibration period of 30 min, perfusion pressure was held constant using a STH Pump Controller (ADInstruments, Bella Vista, NSW, Australia). Once readings were stable, the thromboxane A_2_ mimetic U46619 (10 μmol·L^−1^, 0.01 to 0.1 mL min^−1^) was infused continuously through the aorta using a two‐syringe infusion pump (model sp210iw; World Precision Instruments, SA, Australia) to achieve a 50% reduction in coronary flow rate (i.e., from ~10 to ~5 mL min^−1^). Then, a single bolus dose of vehicle (NaOH; 10 mmol·L^−1^) was administered via an injection port above the aortic cannula, followed by construction of serial concentration–response curves to the NO• donor DEA/NO (10^−10^ to 10^−5^ mol) or the HNO donor Angeli's salt (10^−10^ to 10^−5^ mol) in the presence or absence of 1H‐[1,2,4]oxadiazolo[4,3‐a]quinoxaline‐1‐one (ODQ; 10 μmol·L^−1^, 30 min pre‐incubation, sGC inhibitor) with each bolus dose administered ~1 min apart. Following a washout period of 10 min, a single bolus of Humalog® rapid‐acting insulin (33.3 IU·mL^−1^; Eli Lilly, Indianapolis, IN, USA) was administered via a second injection port above the aortic cannula to assess acute cardiac responses to insulin in non‐diabetic versus diabetic hearts (Figure S2). Three minutes post‐insulin administration, the left ventricle was snap‐frozen for subsequent analysis of protein expression via western blotting. Throughout the experiment, left ventricular systolic pressure (LVSP), left ventricular‐end diastolic pressure (LVEDP), left ventricular developed pressure (LVDP), LV ± dP/dt, coronary flow rate, perfusion pressure, and heart rate were continuously recorded by the PowerLab/LabChart data acquisition system (ADInstruments, Bella Vista, NSW, Australia).

### 2.4 Vascular reactivity ex vivo

Following excision of the heart, the mesenteric arcade was isolated and immediately placed in ice‐cold Krebs' (mmol·L^−1^: 120 NaCl, 5 KCl, 1.2 MgSO_4_, 1.2 KH_2_PO_4_, 25 NaHCO_3_, 11.1 d‐glucose, 2.5 CaCl_2_). Indomethacin (0.01 mmol·L^−1^), a non‐selective cyclooxygenase inhibitor, was added to the Krebs' buffer to prevent synthesis of prostanoids by the artery segments. Small mesenteric arteries (second‐order branches of the superior mesenteric artery) were isolated, cleared of fat and loose connective tissue, cut into rings 2 mm in length, and mounted on a 4 channel Mulvany‐Halpern wire myograph (model 610M; Danish Myo Technology, Aarhus, Denmark). All wire myography experiments were performed at 37°C with organ baths continuously bubbled with carbogen (95% O_2_ and 5% CO_2_). Arteries were allowed to stabilise at zero tension for 30 min prior to normalisation, which was performed by stretching arteries in increments to achieve a final tension equivalent to 70 mmHg, as previously described (Tare et al., 2017). Thirty minutes following normalisation, arteries were maximally contracted by brief exposure to high K^+^ physiological saline solution (KPSS; mmol·L^−1^: 25 NaCl, 100 KCl, 1.2 MgSO_4_, 1.2 KH_2_PO_4_, 25 NaHCO_3_, 11.1 D‐glucose, and 2.5 CaCl_2_). Then, arteries were rinsed and pre‐contracted to 70–80% of their maximal contraction to KPSS using a combination of the thromboxane A_2_ mimetic U46619 (0.1 μmol·L^−1^) and the selective α_1_‐adrenoceptor agonist phenylephrine (0.5 to 5 μmol·L^−1^). Integrity of the endothelium was assessed by exposing the vessel to a single concentration of the endothelium‐dependent vasodilator acetylcholine (ACh; 10 μmol·L^−1^), with relaxation >80% of the pre‐contracted tone accepted as evidence of intact endothelium. Arteries were then washed and precontracted to similar levels (70–80% of KPSS response) using U46619 (0.1 μmol·L^−1^) and phenylephrine (0.5 to 5 μmol·L^−1^), followed by construction of cumulative concentration–response curves to ACh (0.1 nmol·L^−1^ to 50 μmol·L^−1^), the endothelium‐independent vasodilator sodium nitroprusside (SNP; 0.1 nmol·L^−1^ to 50 μmol·L^−1^), the NO• donor DEA/NO (0.1 nmol·L^−1^ to 50 μmol·L^−1^), or the HNO donor Angeli's salt (0.1 nmol·L^−1^ to 50 μmol·L^−1^) (Figure S3). Vasorelaxation evoked by DEA/NO or Angeli's salt was also assessed in the presence or absence of the NO• scavenger hydroxocobalamin (HXC; 100 μmol·L^−1^, 15 min pre‐incubation), the HNO scavenger l‐cysteine (3 mmol·L^−1^, 5 min pre‐incubation) or the sGC inhibitor ODQ (10 μmol·L^−1^, 30 min pre‐incubation).

### 2.5 Western blotting

The immuno‐related procedures used comply with the recommendations made by the *British Journal of Pharmacology* (Alexander et al., 2018). The procedures used with respect to protein analyses were performed in a blinded‐fashion. Left ventricular protein was extracted by homogenisation in RIPA buffer as previously described (Tate et al., 2019). Protein concentration was determined using the BCA protein assay kit (Sigma‐Aldrich). Protein lysates containing an equal amount of protein (30 μg) were loaded into 7.5–12% SDS‐PAGE gels and electrophoresed prior to transferring to a polyvinylidene difluoride membrane (Immobilon‐FL, Millipore). Membranes were blocked with 5% BSA in TBST for 60 min at room temperature and then probed overnight at 4°C with either a rabbit polyclonal (IgG) antibody to detect p‐Akt (when phosphorylated at Ser473; #9271; 1:1000 dilution; Cell Signalling; RRID:AB_329825), which is a mediator of insulin‐stimulated glucose uptake (Muniyappa et al., 2007); a rabbit monoclonal (IgG) antibody to detect endothelial NO• synthase (eNOS) when phosphorylated at Ser1177 (p‐eNOS; ab215717; 1:500 dilution; Abcam; RRID:AB_2893314); or a rabbit polyclonal (IgG) antibody to detect p22^phox^ (sc‐20781; 1:200 dilution; Santa Cruz; RRID:AB_2090309). Membranes that were probed with p‐Akt or p‐eNOS were then stripped using 1× ReBlot Plus Strong solution (10× stock diluted in TBST; Merck, Kenilworth, NJ, USA) and re‐probed with a rabbit polyclonal (IgG) antibody to detect total Akt (#9272; 1:1000 dilution; Cell Signalling; RRID:AB_329827) or a mouse monoclonal (IgG) antibody to detect total eNOS (ab76198; 1:500 dilution; Abcam; RRID:AB_1310183), respectively. All membranes were re‐probed with rabbit polyclonal (IgG) antibody to detect the loading control β‐Actin (#4967; 1:1000 dilution; Cell Signalling; RRID:AB_330288). In each instance, membranes were further probed with goat polyclonal anti‐rabbit HRP‐conjugated secondary antibody (#7074; 1:2000 dilution; Cell Signalling; RRID:AB_2099233). All antibodies were diluted in 5% BSA in TBST and used once. Blots were visualised by chemiluminescence (Western Lighting Plus‐ECL; Perkin Elmer, Waltham, MA, USA) using the ChemiDoc imaging system (Bio‐Rad, Hercules, CA, USA), and signals were quantified by densitometry using Image Lab software (Bio‐Rad).

### 2.6 Mass spectrometry

N‐ethylmaleimide (NEM) derivatised thiol analysis was adapted from Ortmayr et al. (Ortmayr et al., 2015). Briefly, total thiols in left ventricles were extracted using an appropriate amount (10 μL of solvent per 1 mg left ventricular tissue) of extraction solvent: 80% methanol containing 10‐mM ammonium formate at pH 7 and freshly added 25 mM of NEM. Frozen tissue was weighed and transferred to 2‐mL LoBind Eppendorf microcentrifuge tubes on dry ice. Cold extraction solvent was added to samples and tissue was homogenised in a TissueLyser II (Qiagen, Hilden, NW, Germany) for 1 min at 30 s intervals. After vortexing for 30 min at room temperature, samples were centrifuged at 21,000 × *g* for 10 min at room temperature, and supernatant was transferred to vials. Total thiols in plasma were extracted by combining 20 μL of plasma with 200 μL of freshly prepared cold extraction solvent. After vortexing for 30 min at room temperature, samples were centrifuged at 21,000 × *g* for 10 min at room temperature and supernatant was transferred to vials. LCMS data were acquired on Q‐Exactive Orbitrap mass spectrometer (Thermo Scientific, Waltham, MA, USA) coupled with high‐performance liquid chromatography (HPLC) system Dionex Ultimate® 3000 RS (Thermo Scientific). Chromatographic separation was performed on HILIC‐Z column (2.7 μm, 2.1 × 100 mm, Agilent Technologies). The mobile phase (A) was 20‐mM ammonium carbonate and (B) acetonitrile. The gradient started at 90% B and was decreased gradually to 65% B at 10 min, to 20% B at 11.5 min, kept at 20% B until 13 min, brought back to 90% B at 14 min and kept at 90% B until 20 min. A flow rate of 0.3 mL min^−1^ and column compartment temperature of 25°C was used. The total run time was 20 min with an injection volume of 10 μl. Mass spectrometer operated in full scan mode with positive and negative polarity switching at 35,000 resolution at 200 *m*/*z* with a detection range of 85 to 1275 *m*/*z*. Electro‐spray ionisation source (HESI) was set to 3.5‐kV voltage for positive mode and 3.5 kV for negative mode. Sheath gas was set to 50, aux gas to 20 and sweep gas to two arbitrary units, capillary temperature 300°C, probe heater temperature 120°C, S‐Lens RF level 50. Data processing was performed using Tracefinder application (Thermo Scientific) by integrating *m*/*z* of positively charged ions within 5 ppm mass error window. Peak identities were confirmed using reference standards: GSSG [2+] *m*/*z* 307.0832 10.8 min, methionine *m*/*z* 150.0583 5.12 min, NEM‐Cys *m*/*z* 247.0747 4.0 min, NEM‐Cys‐Gly *m*/*z* 304.0962 6.16 min, NEM‐GSH *m*/*z* 433.1388 6.2 min, NEM‐Hcys *m*/*z* 261.0904 4.45 min, NEM‐γ‐Glu‐Cys *m*/*z* 376.1173 5.6 min, SAM *m*/*z* 399.1436 10.8 min, serine *m*/*z* 106.0499 7.8 min.

### 2.7 Statistical analyses

The data and statistical analysis in this study complies with the recommendations on experimental design and analysis in pharmacology. Data are expressed as mean ± SEM and were analysed using GraphPad Prism v8.0 (GraphPad Software, San Diego, CA, USA; RRID:SCR_002798), only if group size was at least *n* = 5, where *n* refers to biological samples and not technical replicates. Changes in haemodynamic parameters in Langendorff‐perfused hearts were measured as the arithmetic difference from baseline (denoted by ∆) following a bolus dose of DEA/NO or Angeli's salt, with the vehicle (10 mmol·L^−1^ NaOH) response subtracted. Dose–response curves and maximal responses (at 10^−5^ mol) in Langendorff‐perfused hearts were analysed using two‐way repeated measures ANOVA. Sidak's post hoc test for multiple comparisons was performed only if the overall ANOVA *P* value achieved statistical significance (i.e., *P* < 0.05), and there was no significant variance in homogeneity. Data comparing cardiac responses to insulin during Langendorff‐perfusion and optical density (OD) values from western blotting were analysed by Student's unpaired *t* test. Cumulative concentration–response curves obtained from mesenteric arteries were computer‐fitted to a sigmoidal curve using nonlinear regression to calculate the potency of each agonist (pEC_50_). Maximum relaxation (R_max_) was expressed as percentage reversal of the level of pre‐contraction to U46619 and phenylephrine. Between group comparisons for pEC_50_ and R_max_ were evaluated by Student's unpaired *t* test. Differences in metabolic characteristics were analysed by two‐way repeated measures ANOVA with Sidak's post hoc test for multiple comparisons, or unpaired Student's *t* test, where appropriate. For peak area data obtained by mass spectrometry, all individual values from each group (including those from the non‐diabetic group) were normalised to the mean of the non‐diabetic group, which was expressed as 1 in each case. This data was analysed using the Mann–Whitney *U* test. No approaches were used to reduce unwanted sources of variation by data normalisation or to generate normal data. Differences were considered statistically significant at *P* < 0.05, and the threshold value was not varied.

### 2.8 Reagents

All drugs were purchased from Sigma‐Aldrich, except for U46619 and ODQ, which were obtained from Cayman Chemical (Ann Arbor, MI, USA). All drugs were prepared in distilled water, except for indomethacin, which was dissolved in 0.1 mol·L^−1^ sodium carbonate, and U46619 and ODQ, which were dissolved in 100% ethanol, with all subsequent dilutions in Krebs. Stock and working solutions of DEA/NO and Angeli's salt were prepared fresh daily in NaOH (10 mmol·L^−1^). All drugs were kept on ice until required.

### 2.9 Nomenclature of targets and ligands

Key protein targets and ligands in this article are hyperlinked to corresponding entries in http://www.guidetopharmacology.org, and are permanently archived in the Concise Guide to PHARMACOLOGY 2021/22 (Alexander et al., 2021).

## 3 RESULTS

### 3.1 Body weights and metabolic characteristics

Fortnightly body weight and blood glucose measurements are displayed in Figure S4. Diabetic rats had a lower body weight during the final 4 weeks of the study (Figure S4A; *P* < 0.05 vs. non‐diabetic rats) and elevated blood glucose levels throughout the entire study post‐STZ administration (Figure S4B; *P* < 0.05 vs. non‐diabetic rats). Glucose tolerance was impaired in diabetic rats, indicated by a greater area under the curve (AUC; normalised to baseline blood glucose level), and failure for blood glucose levels to return to baseline at test completion (Figure S4C; *P* < 0.05 vs. non‐diabetic rats). During the insulin tolerance test, diabetic rats displayed elevated blood glucose levels at baseline, which declined considerably 30 min post‐insulin administration, but did not reach those of non‐diabetic rats, and remained elevated at test completion (Figure S4D). At study end‐point (12 weeks post‐vehicle or STZ), glycated haemoglobin, and plasma insulin and triglycerides were elevated in the diabetic group (Table S1; *P* < 0.05 vs. non‐diabetic rats). Plasma levels of total cholesterol did not differ between groups (Table S1). However, HDL levels were lower, and the total cholesterol to HDL ratio and LDL/VLDL to HDL ratio was higher in diabetic rats (Table S1; *P* < 0.05 vs. non‐diabetic rats).

### 3.2 Baseline haemodynamic parameters in Langendorff‐perfused hearts

There were no significant differences in pressure values at baseline following equilibration, after pre‐treatment with ODQ or during U46619 infusion between non‐diabetic and diabetic Langendorff‐perfused hearts (Table S2). Heart rate was lower in diabetic hearts, when compared with non‐diabetic hearts (Table S2; *P* < 0.05 vs. non‐diabetic group).

### 3.3 Insulin signalling and expression of eNOS and p22^phox^ in Langendorff‐perfused hearts

**FIGURE 1:**
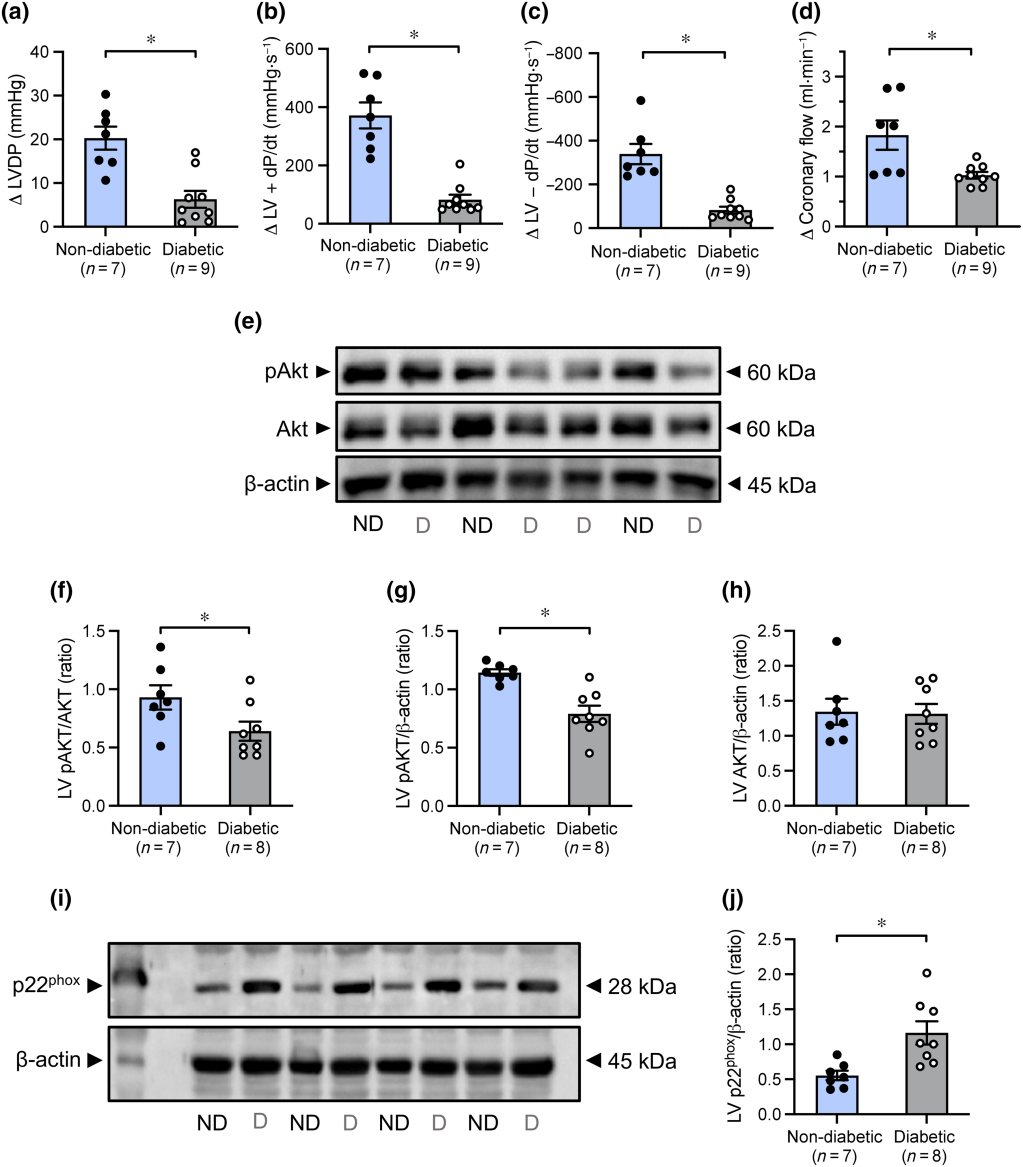
Change in (a) LVDP, (b) LV + dP/dt, (c) LV − dP/dt, and (d) coronary flow rate, in addition to LV protein expression of (f) phospho‐Akt/total‐Akt, (g) phospho‐Akt/β‐actin, (h) total‐Akt/β‐actin, and (j) p22^phox^/β‐actin following a bolus dose of insulin in non‐diabetic and diabetic Langendorff‐perfused hearts. Representative LV immunoblot of (e) phospho‐Akt, total‐Akt and β‐actin, and (i) p22^phox^ and β‐actin. Uncropped blots are provided in Figures S7 and S9. Data are presented as mean ± SEM. Data analysed by Student's unpaired *t* test. *P < 0.05 compared with the non‐diabetic group. ∆, change from baseline; LV, left ventricular; LVDP, LV developed pressure; LV + dP/dt, maximal rate of rise in LV pressure; LV − dP/dt, maximal rate of fall in LV pressure; ND, non‐diabetic; D, diabetic

Reduced cardiac responsiveness to insulin was evident in diabetic hearts, as demonstrated by smaller increases in LVSP, LVEDP (Figure S5A,B), LVDP, LV + dP/dt, LV − dP/dt, and coronary flow rate responses (Figure 1a–d; *P* < 0.05 vs. non‐diabetic hearts) following a bolus dose of insulin, which was accompanied by a diminution in left ventricular phosphorylation of Akt (Figure 1f,g; *P* < 0.05 vs. non‐diabetic group). Heart rate was not altered in response to a bolus dose of insulin in non‐diabetic or diabetic hearts (Figure S5C). There was no difference in left ventricular protein levels of phosphorylated or total eNOS (Figure S6A–C) between non‐diabetic and diabetic hearts. However, p22^phox^ protein expression was higher in left ventricles from diabetic hearts, compared with non‐diabetic hearts (Figure 1j; *P* < 0.05 vs. non‐diabetic group).

### 3.4 Impact of diabetes on cardiac inotropic and lusitropic responses to exogenous NO•/HNO

**FIGURE 2:**
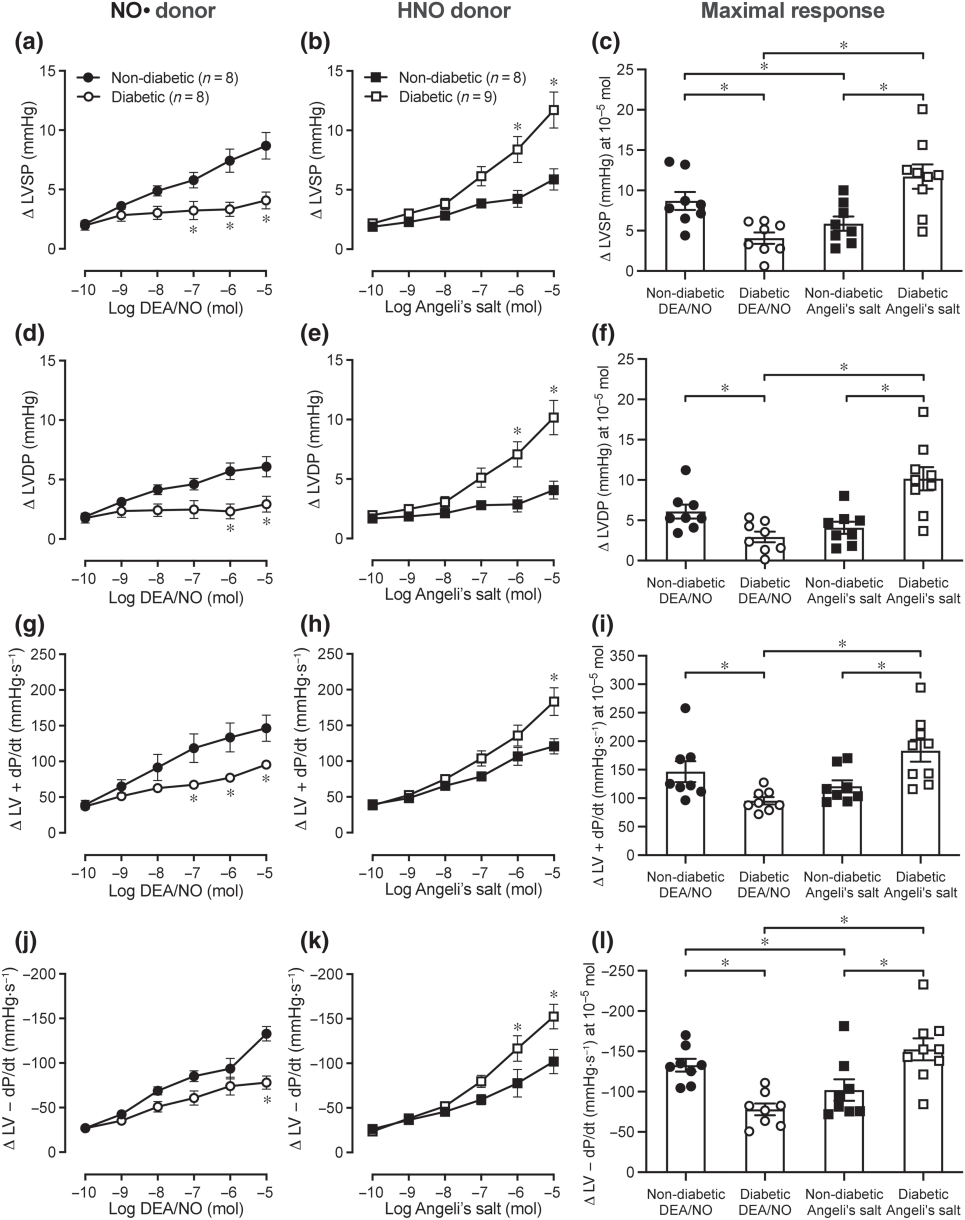
Dose–response curves and maximal responses to the NO*•* donor DEA/NO or the HNO donor Angeli's salt in Langendorff‐perfused hearts. (a–c) LVSP, (d–f) LVDP, (g–i) LV + dP/dt, and (j–l) LV − dP/dt in non‐diabetic and diabetic hearts. Data are expressed as change from baseline (denoted by ∆), mean ± SEM. Data analysed by two‐way repeated measures ANOVA with Sidak's post hoc test for multiple comparisons. *P < 0.05. NO*•*, nitric oxide; HNO, nitroxyl; LV, left ventricular; LVSP, LV systolic pressure; LVDP, LV developed pressure; LV + dP/dt, maximal rate of rise in LV pressure; LV − dP/dt, maximal rate of fall in LV pressure

In hearts isolated from diabetic rats, positive inotropic responses to DEA/NO (i.e., exogenous NO*•*) were markedly attenuated, indicated by smaller increases in LVSP (Figure 2a,c; *P* < 0.05 vs. non‐diabetic hearts), LVDP (Figure 2d; *P* < 0.05 vs. non‐diabetic hearts) and LV + dP/dt (Figure 2g; *P* < 0.05 vs. non‐diabetic hearts) responses. However, the HNO donor, Angeli's salt, caused dose‐dependent increases in LVSP (Figure 2b,c; *P* < 0.05 vs. non‐diabetic hearts), LVDP (Figure 2e,f; *P* < 0.05 vs. non‐diabetic hearts), and LV + dP/dt (Figure 2h; *P* < 0.05 vs. non‐diabetic hearts) that were enhanced in diabetic hearts. Similarly, lusitropic responses measured by LV − dP/dt were lower in response to DEA/NO in diabetic hearts (Figure 2j,l; *P* < 0.05 vs. non‐diabetic hearts), but enhanced in response to Angeli's salt (Figure 2k; *P* < 0.05 vs. non‐diabetic hearts). DEA/NO elicited dose‐dependent increases in LVEDP in non‐diabetic hearts (Figure S10A,C), which did not reach statistical significance in response to Angeli's salt in non‐diabetic or diabetic hearts (Figure S10B,C).

### 3.5 Impact of diabetes on coronary vasodilation and heart rate in response to exogenous NO•/HNO

**FIGURE 3:**
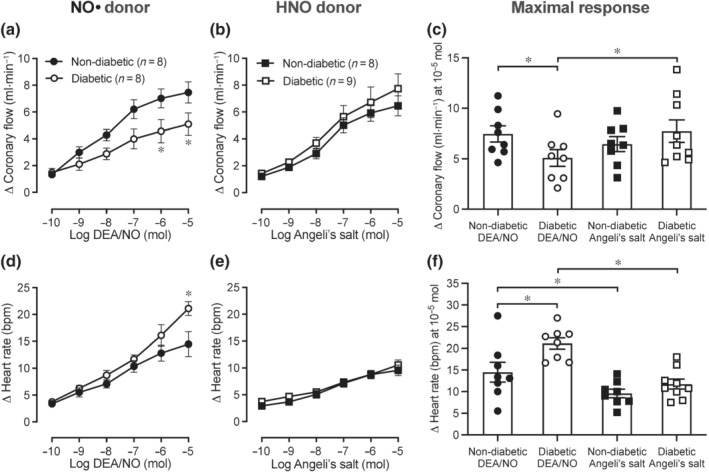
Dose–response curves and maximal responses to NO*•* donor DEA/NO or HNO donor Angeli's salt in Langendorff‐perfused hearts. (a–c) Coronary flow rate and (d–f) heart rate in response to DEA/NO or Angeli's salt in non‐diabetic and diabetic hearts. Data are expressed as change from baseline (denoted by ∆), mean ± SEM. Data analysed by two‐way repeated measures ANOVA with Sidak's post hoc test for multiple comparisons. *P < 0.05. NO*•*, nitric oxide; HNO, nitroxyl

As shown in Figure 3, DEA/NO and Angeli's salt both caused dose‐dependent increases in coronary flow rate in non‐diabetic hearts (Figure 3a,b). Conversely, the coronary flow response to DEA/NO was attenuated in diabetic hearts (Figure 3a; *P* < 0.05 vs. non‐diabetic hearts), but was maintained (Figure 3b) or enhanced (Figure 3c; *P* < 0.05 vs. diabetic DEA/NO) in response to Angeli's salt. In diabetic hearts, DEA/NO also elicited dose‐dependent increases in heart rate (Figure 3d,f; *P* < 0.05 vs. non‐diabetic hearts), which was not evident in response to Angeli's salt (Figure 3e,f).

### 3.6 Role of sGC in inotropic and lusitropic responses to exogenous NO•/HNO

**FIGURE 4:**
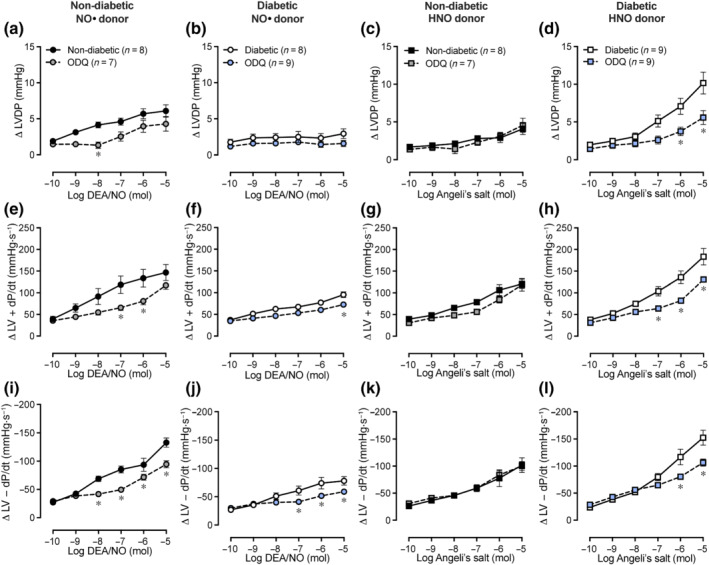
Impact of ODQ on dose–response curves to DEA/NO or Angeli's salt in non‐diabetic and diabetic Langendorff‐perfused hearts. Increases in LVDP response to DEA/NO in (a) non‐diabetic and (b) diabetic hearts, or Angeli's salt in (c) non‐diabetic and (d) diabetic hearts. Increases in LV + dP/dt response to DEA/NO in (e) non‐diabetic and (f) diabetic hearts, or Angeli's salt in (g) non‐diabetic and (h) diabetic hearts. Increases in LV − dP/dt response to DEA/NO in (i) non‐diabetic and (j) diabetic hearts, or Angeli's salt in (k) non‐diabetic and (l) diabetic hearts. Data are expressed as change from baseline (denoted by ∆), mean ± SEM. Data analysed by two‐way repeated measures ANOVA with Sidak's post hoc test for multiple comparisons. *P < 0.05 compared with absence of ODQ. NO*•*, nitric oxide; HNO, nitroxyl; LV, left ventricular; LVDP, LV developed pressure; LV + dP/dt, maximal rate of rise in LV pressure; LV − dP/dt, maximal rate of fall in LV pressure; ODQ, 1*H*‐[1,2,4]oxadiazolo[4,3‐a]quinoxaline‐1‐one (sGC inhibitor)

Incubation with the sGC inhibitor, ODQ, led to inhibition of positive inotropic responses to DEA/NO, as indicated by attenuated increases in LVDP and LV + dP/dt (Figure 4a,e; *P* < 0.05 vs. absence of ODQ) in non‐diabetic hearts. However, ODQ did not change the response to the HNO donor, Angeli's salt, on LVSP (Figure S11C), LVDP and LV + dP/dt (Figure 4c,g) in non‐diabetic hearts. In contrast, in diabetic hearts, ODQ blunted increases in LVSP (Figure S11D; *P* < 0.05 vs. absence of ODQ), LVDP, and LV + dP/dt (Figure 4d,h; *P* < 0.05 vs. absence of ODQ) to Angeli's salt. In the presence of ODQ, lusitropic responses to DEA/NO were also blunted, indicated by smaller increases in LV − dP/dt in non‐diabetic (Figure 4i; *P* < 0.05 vs. absence of ODQ) and diabetic hearts (Figure 4j; *P* < 0.05 vs. absence of ODQ). ODQ also caused a rightward shift in the dose–response curve for LV − dP/dt in response to Angeli's salt in diabetic hearts (Figure 4l; *P* < 0.05 vs. absence of ODQ), but no significant change was evident in non‐diabetic hearts (Figure 4k). There were no significant differences in the increases in LVEDP in response to DEA/NO in the presence of ODQ in non‐diabetic (Figure S11E) and diabetic hearts (Figure S11F). Similarly, increases in LVEDP in response to Angeli's salt were not significantly altered by ODQ in non‐diabetic hearts (Figure S11G). In contrast, ODQ attenuated increases in LVEDP in response to Angeli's salt in diabetic hearts (Figure S11H; *P* < 0.05 vs. absence of ODQ).

### 3.7 Role of sGC in coronary vasodilation and heart rate responses to exogenous NO•/HNO

**FIGURE 5:**
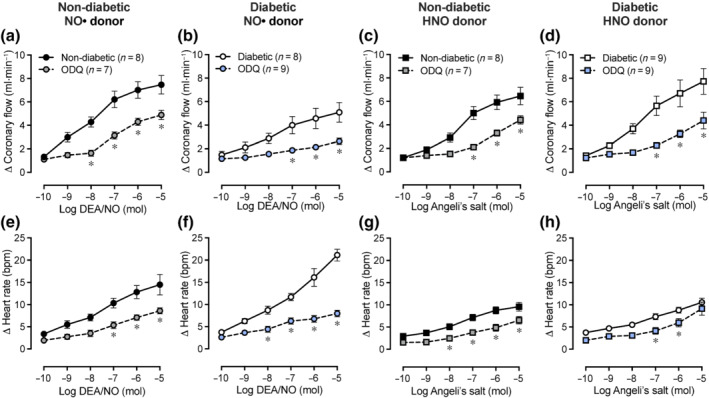
Impact of ODQ on dose–response curves to DEA/NO or Angeli's salt in non‐diabetic and diabetic Langendorff‐perfused hearts. Increases in coronary flow rate response to DEA/NO in (a) non‐diabetic and (b) diabetic hearts, or Angeli's salt in (c) non‐diabetic and (d) diabetic hearts. Increases in heart rate response to DEA/NO in (e) non‐diabetic and (f) diabetic hearts, or Angeli's salt in (g) non‐diabetic and (h) diabetic hearts. Data are expressed as change from baseline (denoted by ∆), mean ± SEM. Data analysed by two‐way repeated measures ANOVA with Sidak's post hoc test for multiple comparisons. *P < 0.05 compared with absence of ODQ. NO*•*, nitric oxide; HNO, nitroxyl; ODQ, 1*H*‐[1,2,4]oxadiazolo[4,3‐a]quinoxaline‐1‐one (sGC inhibitor)

In the presence of ODQ, the increase in coronary flow rate in response to DEA/NO or Angeli's salt was blunted in non‐diabetic (Figure 5a,c; *P* < 0.05 vs. absence of ODQ) and diabetic hearts (Figure 5b,d; *P* < 0.05 vs. absence of ODQ). The increases in heart rate in response to DEA/NO or Angeli's salt were blunted by ODQ in non‐diabetic and diabetic hearts (Figure 5e–h; *P* < 0.05 vs. absence of ODQ).

### 3.8 Metabolites of the methionine and glutathione pathways, and the glutathione redox cycle

**FIGURE 6:**
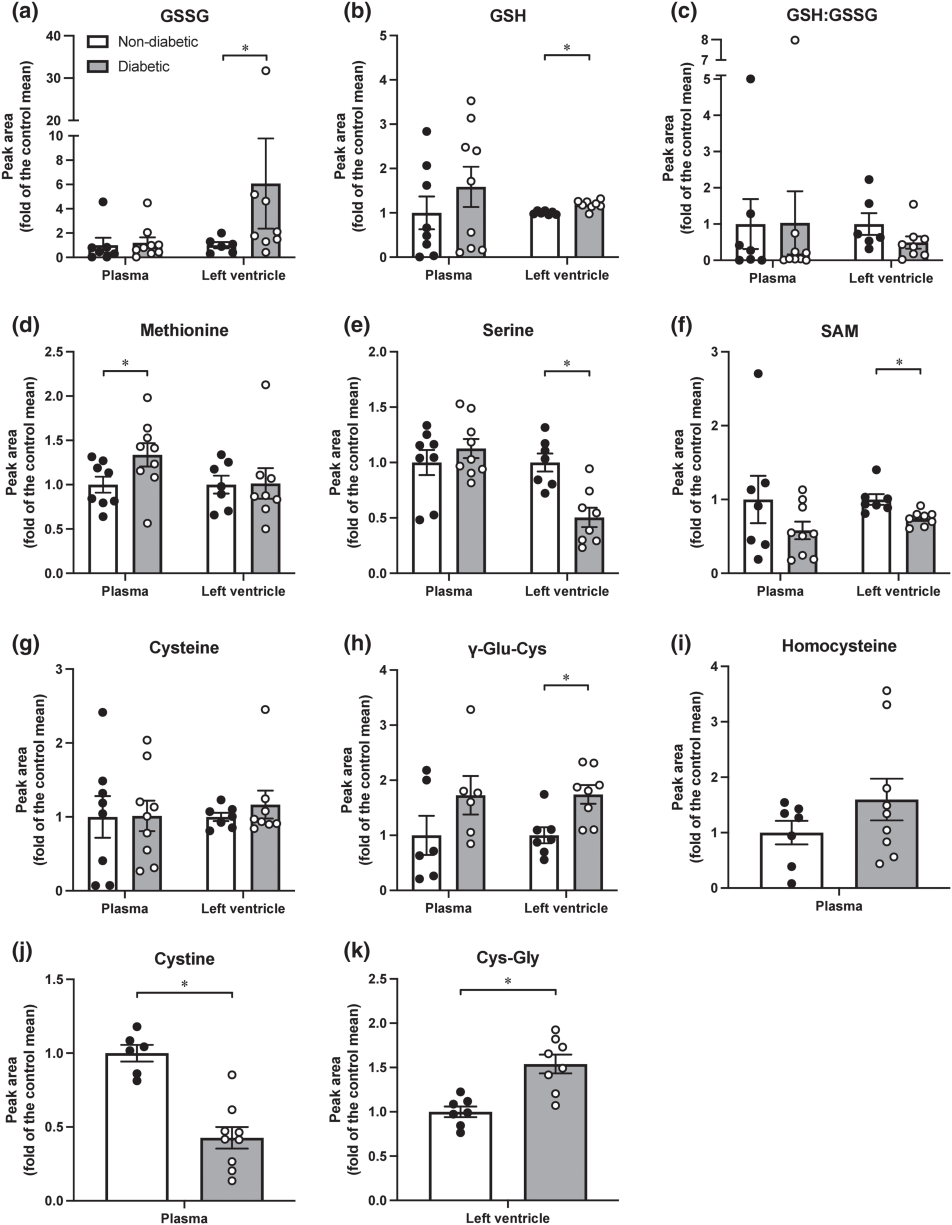
Impact of diabetes on metabolites of the methionine and glutathione pathway, and the glutathione redox cycle. Peak area of (a) GSSG, (b) GSH, (c) GSH:GSSG, (d) methionine, (e) serine, (f) SAM, (g) cysteine, and (i) γ‐Glu‐Cys in plasma and left ventricles from non‐diabetic and diabetic rats. Peak area of (i) homocysteine and (j) cystine in plasma from non‐diabetic and diabetic rats. Peak area of (k) Cys‐Gly in left ventricles from non‐diabetic and diabetic rats. Values are expressed as mean ± SEM (relative to that of the non‐diabetic group, which was expressed as 1 in each case). *N* = 6–9 animals. Data analysed by the Mann–Whitney *U* test. *P < 0.05 compared with the non‐diabetic group. GSSG, oxidised glutathione; GSH, reduced glutathione; SAM, *S*‐adenosyl‐methionine; γ‐Glu‐Cys, γ‐glutamylcysteine; Cys‐Gly, cysteinylglycine

In left ventricles from Langendorff‐perfused hearts, levels of oxidised glutathione (GSSG) and reduced glutathione (GSH) were elevated in the diabetic group (Figure 6a,b; *P* < 0.05 vs. non‐diabetic group). There were no significant differences in plasma levels of GSSG or GSH between groups (Figure 6a,b). The GSH:GSSG ratio tended to be lower in left ventricles from diabetic hearts, although this did not reach statistical significance (Figure 6c; *P* = 0.1 vs. non‐diabetic group). Plasma levels of methionine were higher in the diabetic group (Figure 6d; *P* < 0.05 vs. non‐diabetic group). However, left ventricular levels of methionine did not differ between groups (Figure 6d). Levels of serine and *S*‐adenosyl‐methionine (SAM) were lower in left ventricles from diabetic rats (Figure 6e,f; *P* < 0.05 vs. non‐diabetic group) but were not different in plasma from these animals (Figure 6e,f). There were no significant differences in cysteine levels in plasma or left ventricles from non‐diabetic or diabetic rats (Figure 6g). In the diabetic group, γ‐glutamylcysteine (γ‐Glu‐Cys) was elevated in left ventricles (Figure 6h; *P* < 0.05 vs. non‐diabetic group), but not plasma (Figure 6h). Plasma levels of homocysteine did not differ between groups (Figure 6i), whereas cystine was lower in the diabetic group (Figure 6j; *P* < 0.05 vs. non‐diabetic group). Cysteinylglycine (Cys‐Gly) was elevated in left ventricles from diabetic hearts (Figure 6k; *P* < 0.05 vs. non‐diabetic group).

### 3.9 Relaxation responses in mesenteric arteries

**FIGURE 7:**
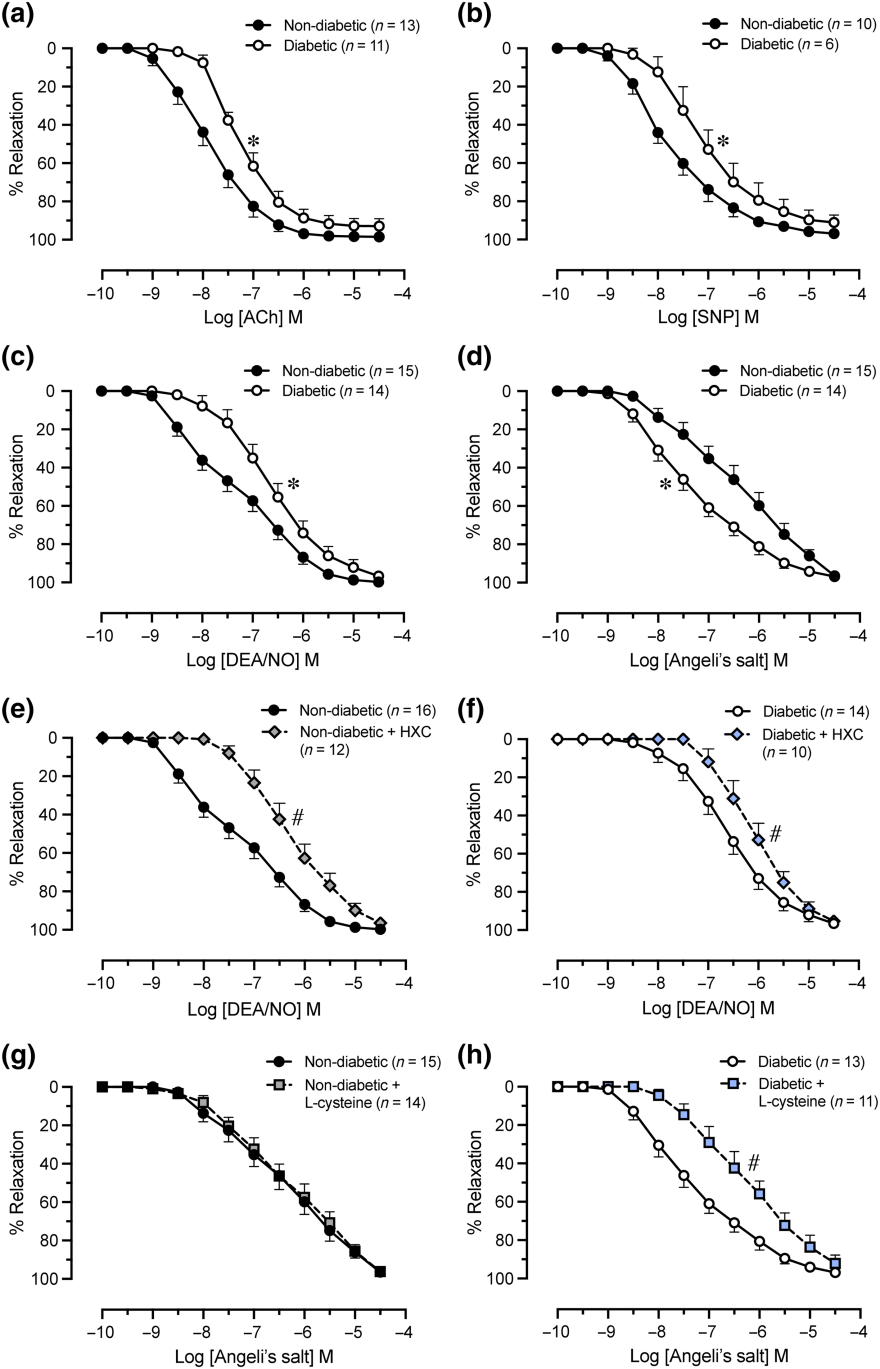
Cumulative concentration–response curves to (a) ACh, (b) SNP, (c) DEA/NO in the absence or (e, f) presence of the NO• scavenger HXC (100 μmol·L^−1^), and Angeli's salt in the (d) absence or (g, h) presence of the HNO scavenger l‐cysteine (3 mmol·L^−1^) in non‐diabetic and diabetic mesenteric arteries. Values are expressed as % reversal of pre‐contraction and given as mean ± SEM, *n* = number of animals. Potency (pEC_50_) and maximum relaxation (R_max_) values are provided in Table 1. *P < 0.05, pEC_50_ compared with the non‐diabetic group. ^#^ *P* < 0.05, pEC_50_ compared with absence of scavenger. ACh, acetylcholine; SNP, sodium nitroprusside; HXC, hydroxocobalamin

We first explored the impact of diabetes on endothelium‐dependent and endothelium‐independent relaxation responses in mesenteric arteries. Relaxation in response to the endothelium‐dependent vasodilator ACh was impaired in arteries from diabetic rats (Table 1 and Figure 7a; *P* < 0.05 vs. non‐diabetic arteries). Diabetes was also associated with impaired SNP‐induced endothelium‐independent vasorelaxation (Table 1 and Figure 7b; *P* < 0.05 vs. non‐diabetic arteries). There was however no significant difference in the maximal relaxation response to ACh or SNP between non‐diabetic and diabetic arteries (Table 1). Next, we investigated the impact of diabetes on relaxation responses to DEA/NO and Angeli's salt in mesenteric arteries. Relaxation to the NO• donor DEA/NO was lower in arteries from diabetic rats (Table 1 and Figure 7c; *P* < 0.05 vs. non‐diabetic arteries). In contrast, relaxation to the HNO donor Angeli's salt was enhanced (Table 1 and Figure 7d; *P* < 0.05 vs. non‐diabetic arteries). However, the maximal relaxation response to DEA/NO or Angeli's salt did not differ significantly between non‐diabetic and diabetic arteries (Table 1). We then examined the effect of pharmacological inhibitors on relaxation to DEA/NO and Angeli's salt in mesenteric arteries. The NO• scavenger, HXC, caused a rightward shift in the concentration–response curve to DEA/NO in arteries from non‐diabetic (Table 1 and Figure 7f; *P* < 0.05 vs. absence of HXC) and diabetic rats (Table 1 and Figure 7f; *P* < 0.05 vs. absence of HXC). In contrast, HXC did not significantly attenuate Angeli's salt‐mediated relaxation in arteries from non‐diabetic or diabetic rats (Figure S12A,B). Conversely, vasorelaxation to Angeli's salt was not attenuated by the HNO scavenger, l‐cysteine, in non‐diabetic arteries (Table 1 and Figure 7g) but induced substantial inhibition of relaxation to Angeli's salt in diabetic arteries (Table 1 and Figure 7h; *P* < 0.05 vs. absence of l‐cysteine). The sGC inhibitor, ODQ, attenuated vasorelaxation to Angeli's salt in arteries from non‐diabetic (Figure S12C) and diabetic rats (Figure S12D). In a subset of vessels, we also obtained pilot data suggesting that the rightward shift caused by ODQ in the concentration–response curve to Angeli's salt was greater in non‐diabetic arteries, when compared with diabetic arteries (Figure S12E), although, firm conclusions cannot be drawn from this small subset.

**TABLE 1:** Relaxation to endothelium‐dependent and endothelium‐independent vasodilators in mesenteric arteries from non‐diabetic and diabetic rats

	Non‐diabetic			Diabetic		
	*n*	pEC_50_	R_max_	*n*	pEC_50_	R_max_
ACh	13	7.89 ± 0.17	99 ± 0	11	7.27 ± 0.10*	93 ± 4
SNP	10	7.79 ± 0.17	97 ± 1	6	7.14 ± 0.24*	91 ± 4
DEA/NO	15	7.34 ± 0.18	99 ± 0	14	6.69 ± 0.20*	97 ± 1
DEA/NO + HXC	12	6.44 ± 0.23**	97 ± 1	10	6.10 ± 0.18**	95 ± 1
Angeli's salt	15	6.63 ± 0.24	97 ± 1	14	7.37 ± 0.18*	97 ± 1
Angeli's salt + L‐cysteine	14	6.48 ± 0.22	96 ± 1	11	6.44 ± 0.22***	92 ± 4

*Note*: Values are expressed as mean ± SEM. pEC_50_ values are expressed as −log M. R_max_ values are expressed as % reversal of pre‐contraction. Data analysed by Student's unpaired *t* test.

Abbreviations: ACh, acetylcholine; HXC, hydroxocobalamin; *n*, number of animals; pEC_50_, potency; R_max_, maximum relaxation; SNP, sodium nitroprusside.

* *P* < 0.05 compared with the non‐diabetic group.

** *P* < 0.05 compared with DEA/NO.

*** *P* < 0.05 compared with Angeli's salt.

## 4 DISCUSSION

This is the first report that the myocardium is susceptible to T2DM‐induced NO• resistance, as has been previously reported for human platelets (Anderson et al., 2005; Worthley et al., 2007). Furthermore, we confirm that there was no evidence of T2DM‐induced resistance to HNO in the myocardium, and that the reduced responsiveness to NO• in T2DM myocardium could be circumvented by HNO. In hearts isolated from this pre‐clinical rat model of T2DM, inotropic, lusitropic and coronary vasodilator responses to the NO• donor DEA/NO were impaired, whereas responses to the HNO donor Angeli's salt were preserved or enhanced. Furthermore, vasorelaxation to Angeli's salt was enhanced in small, diabetic mesenteric arteries, which were hyporesponsive to the vasodilator effects of DEA/NO (Figure S13). The presence of NO• resistance in the coronary vasculature represents an independent risk factor for adverse cardiovascular outcomes (e.g. myocardial infarction) in patients with diabetes presenting with acute coronary syndromes (Schachinger et al., 2000). The findings of this study are therefore of clinical importance, as they highlight the ability of HNO donors to improve cardiac contraction and relaxation, and promote vasodilation, through circumvention of NO• resistance in the diabetic heart and vasculature.

The increase in cardiac contraction and coronary flow rate observed in response to insulin in non‐diabetic hearts, and reduced response in diabetic hearts, is consistent with results of previous studies (Jagasia et al., 2001; Sasso et al., 2000). Sasso et al., identified that insulin infusion increases LVEF and peak filling rate in healthy participants compared with sex‐, age‐, and BMI‐matched diabetic subjects (Sasso et al., 2000). The authors also found that LVEF was positively correlated with the insulin sensitivity index in diabetic subjects, suggesting that cardiac insulin resistance was responsible for the reduction in LVEF and peak filling rate following insulin infusion (Sasso et al., 2000). Similarly, in a study by Jagasia et al., intracoronary insulin infusion increased coronary blood flow in non‐diabetic subjects, but lacked efficacy in those with diabetes (Jagasia et al., 2001). Previous studies examining cardiac insulin signalling suggest that insulin exerts positive inotropic effects through several phosphoinositide 3‐kinase (PI3K)‐dependent mechanisms, some of which include activation of L‐type Ca^2+^ channels and myofilament Ca^2+^ sensitisation (Maier et al., 1999; Von Lewinski et al., 2005). Insulin also induces vasodilation by signalling through the PI3K/Akt pathway, leading to downstream activation of eNOS and subsequent NO• generation (Muniyappa et al., 2007). Given that we identified Akt phosphorylation to be lower in left ventricles from diabetic hearts following a bolus dose of insulin during Langendorff‐perfusion, this indicates that the cardiovascular actions of insulin were blunted in diabetic hearts due to impaired insulin signalling and therefore cardiac insulin resistance.

Inotropic, lusitropic and coronary vasodilator responses to NO• were impaired, but those to HNO were preserved or enhanced in the insulin‐resistant diabetic rat myocardium. At baseline, heart rate was slower in the diabetic group, however pressure values were not different between groups. These observations are consistent with those observed in T1DM rat hearts (Qin et al., 2020). The impairment of DEA/NO‐induced inotropic, lusitropic, and coronary vasodilator responses we observed in diabetic hearts suggests that NO• resistance was present in both the myocardium and the coronary vasculature. These data are the first to show that myocardial NO• resistance is associated with T2DM, adding to previous clinical data demonstrating resistance to the anti‐aggregatory (Anderson et al., 2005; Worthley et al., 2007) and vasodilator (Williams et al., 1996) actions of NO• in patients with T2DM. It should be mentioned that a component of the inotropic actions we observed in response to DEA/NO, which are sGC‐dependent, may be attributed to the Gregg effect, whereby an increase in coronary flow rate leads to an increase in contractile parameters (Westerhof et al., 2006). Thus, it is possible that the attenuation of positive inotropic effect of DEA/NO in diabetic hearts is secondary to the reduced induction of coronary vasodilatation, rather than a direct effect on the myocardium.

NO• resistance occurs as a consequence of oxidative stress whereby elevations in ROS, such as superoxide, leads to scavenging of NO• and oxidation of the ferrous (Fe^2+^) haem group on its intracellular receptor, sGC, to its ferric (Fe^3+^) NO•‐insensitive state (Pacher et al., 2007). We have demonstrated in our previous work that superoxide generated by pyrogallol induces NO• resistance by impairing the ability of the NO• donor DEA/NO to stimulate the activity of purified sGC (Irvine et al., 2013). Oxidative stress is a characteristic of diabetes that has been previously identified in the myocardium of T1DM and T2DM rats (Hamblin et al., 2007; Qin et al., 2020; Zhou et al., 2010). We observed elevated protein levels of p22^phox^ in left ventricles from diabetic Langendorff‐perfused hearts. p22^phox^ plays an important role in oxidative stress by mediating superoxide production by NADPH oxidase (Nox) enzymes 1 and 2 (Drummond & Sobey, 2014). Although we did not measure superoxide levels in Langendorff‐perfused hearts, an elevation in p22^phox^ expression suggests that superoxide generation by Nox1 and 2 may have been up‐regulated. Further, our observations at the level of GSH/GSSG suggests an environment in which an imbalance in the relative proportions of reduced versus oxidised sGC may be present in the diabetic rat heart. Therefore, it is possible that scavenging of NO• by superoxide, and/or oxidation of sGC due to concomitant oxidative stress, are contributing factors to the NO• resistance observed in the diabetic myocardium and coronary vasculature.

Consistent with previous reports (Leo et al., 2012; Qin et al., 2020; Tare et al., 2017), the vasodilator response to Angeli's salt was preserved in the coronary vasculature in diabetic hearts. This is attributed to the ability of HNO to remain effective in the presence of oxidative stress, as it fails to react with superoxide (Leo et al., 2012). Interestingly, the positive inotropic and lusitropic effects of Angeli's salt appear to be enhanced in the diabetic myocardium. A possible explanation for the enhanced response to Angeli's salt could be a reduction in thiol availability in diabetic hearts. HNO has high reactivity with thiols. Previous studies have reported that GSH levels are lower, and GSSG levels higher, in atrial tissue from patients with T2DM (Anderson et al., 2009) and left ventricles from diabetic rats (El‐Seweidy et al., 2011; Xu et al., 2002). In isolated rat cardiomyocytes, increasing intracellular thiol content quenches HNO and blunts the contractile response to Angeli's salt (Tocchetti et al., 2007). Based on this evidence, a reduction in intracellular thiol content could lead to an enhanced response to Angeli's salt. GSH is the most abundant intracellular thiol and functions as an antioxidant by scavenging ROS (e.g., superoxide) (Anderson et al., 2009; Aquilano et al., 2014). We measured metabolites of the methionine and GSH pathways, and the GSH redox cycle (Figure S15) to determine whether levels of GSH, GSSG and other thiols were altered by diabetes in left ventricles from Langendorff‐perfused hearts. In the present study, both GSH (albeit modestly) and GSSG were higher in left ventricles from diabetic rats, suggesting that GSH production was increased to counteract elevated ROS generation (indicated by increased oxidation of GSH to GSSG). Although our observation of a modest increase in diabetic left ventricular GSH contrasts to the decrease previously observed in left ventricles from diabetic rats (El‐Seweidy et al., 2011; Xu et al., 2002), we measured GSH and GSSG directly via mass spectrometry, rather than indirectly via less‐sensitive, colorimetric spectrophotometric approaches (where GSH is calculated as the difference between total glutathione and GSSG).

A reduction in the ratio between the reduced and oxidised form of glutathione (GSH/GSSG) is an indicator of redox imbalance. We observed that the GSH/GSSG ratio tended to be lower in left ventricles from diabetic (compared with non‐diabetic) rats, although this did not reach statistical significance. The thiols γ‐Glu‐Cys and Cys‐Gly were elevated in left ventricles from diabetic hearts, and levels of cysteine remained unchanged across groups. This outcome suggests that the enhanced response to Angeli's salt in diabetic hearts was not attributed to reduced thiol levels. However, insulin administration following completion of dose–response curves during Langendorff‐perfusion may have influenced thiol levels, as it has previously been shown that insulin normalises glutathione levels in cardiomyocytes from T1DM rats (Li et al., 2007). Although it is unclear why responses to Angeli's salt were enhanced in the diabetic myocardium, this observation is consistent with our previous findings in the T1DM myocardium (Qin et al., 2020). Further studies are required to elucidate the mechanism underlying this observation.

The contribution of sGC to inotropic, lusitropic, and coronary vasodilator effects of DEA/NO and Angeli's salt was examined using ODQ, which irreversibly inhibits sGC by oxidising its ferrous (Fe^2+^) heme group to its ferric (Fe^3+^) state (Zhao et al., 2000). DEA/NO‐induced inotropic and lusitropic responses were attenuated by ODQ to a greater extent in non‐diabetic than diabetic hearts, suggesting that sGC‐dependent signalling was impaired in the diabetic myocardium. Administration of ODQ attenuated Angeli's salt‐induced inotropic and lusitropic responses in diabetic hearts, indicating that these effects of HNO are mediated, in part, via sGC‐dependent mechanisms. In contrast, inotropic and lusitropic responses to Angeli's salt were not attenuated by ODQ in non‐diabetic hearts. Such findings may indicate that HNO is able to activate sGC in its oxidised form; however, this remains a matter of contention due to conflicting evidence that either supports this hypothesis (Dautov et al., 2013; Miranda et al., 2003; Qin et al., 2020) or refutes it (Miller et al., 2009; Zeller et al., 2009). We also demonstrated that ODQ attenuated coronary vasodilation in response to Angeli's salt in non‐diabetic and diabetic hearts. In accordance with previous studies (Chin et al., 2014; Favaloro & Kemp‐Harper, 2007; Qin et al., 2020), these results suggest that the vasodilator effects of HNO in the coronary vasculature are mediated predominantly via sGC‐dependent signalling.

Consistent with previous reports, we demonstrated that sensitivity to the endothelium‐dependent vasodilator, ACh, was decreased in mesenteric arteries from diabetic rats (Leo et al., 2011; Tare et al., 2017; Wigg et al., 2001). Impaired vascular responsiveness to the endothelium‐independent NO• donor, SNP, has been identified in the brachial (van Etten et al., 2002; Williams et al., 1996) artery in patients with T2DM. Indeed, in the present study, endothelium‐independent relaxation in response to NO• donors SNP and DEA/NO was attenuated in mesenteric arteries from diabetic rats. These findings are indicative of decreased vascular smooth muscle responsiveness to NO• and thus the presence of NO• resistance. Another important finding was that Angeli's salt circumvented NO• resistance by enhancing vasorelaxation in mesenteric arteries from diabetic rats. In accordance with the current results, previous studies have demonstrated that vascular smooth muscle responsiveness to HNO is preserved in diabetes (Leo et al., 2012; Qin et al., 2020; Tare et al., 2017). Indeed, the superoxide generator, pyrogallol, decreases vascular sensitivity to DEA/NO, but does not alter vasorelaxation to Angeli's salt in the rat aorta (Leo et al., 2012).

Angeli's salt releases equivalent amounts of HNO and nitrite under physiological conditions, however, in comparison with Angeli's salt, nitrite is 15,000‐fold less potent as a vasodilator of mesenteric arteries (Favaloro & Kemp‐Harper, 2007) suggesting the predominant effect of HNO. Moreover, neither nitrite nor degraded Angeli's salt can replicate the cGMP‐elevating and anti‐hypertrophic effects of Angeli's salt in cardiomyocytes (Lin et al., 2012). In the current study, the HNO scavenger l‐cysteine was also utilised to rule out potential vasodilator effects of nitrite co‐released by Angeli's salt in mesenteric arteries. Vasorelaxation to Angeli's salt was markedly attenuated by l‐cysteine in diabetic arteries, confirming that the vasorelaxant actions of Angeli's salt were predominantly mediated by HNO in the diabetic mesenteric vasculature. Unexpectedly, l‐cysteine did not attenuate Angeli's salt‐induced vasorelaxation in non‐diabetic arteries. A possible explanation for the lack of inhibitory action of l‐cysteine in the non‐diabetic vasculature could be the absence of EDTA in the Krebs' buffer. EDTA is a Cu^2+^‐chelator that prevents the oxidation of HNO to NO by Cu^2+^‐containing enzymes, and when included in Krebs' buffer, reduces the amount of NO• generated by Angeli's salt in mesenteric arteries (Irvine et al., 2003). We performed additional wire myography experiments in mesenteric arteries from naïve rats (aged 8–10 weeks) to evaluate whether inclusion or exclusion of EDTA in Krebs' buffer influences relaxation responses to Angeli's salt in the presence or absence of l‐cysteine (Methods S1 and Results S2). In the presence of EDTA, l‐cysteine caused a rightward shift in the concentration–response curve to Angeli's salt. This inhibitory effect of l‐cysteine on relaxation to Angeli's salt was not evident when EDTA was excluded from the Krebs' buffer (Figure S14). It appears, therefore, that inclusion of EDTA in the Krebs' buffer prevents conversion of HNO to NO•. The reason for the observed lack of a detectable NO• component of effect in the presence of HXC in the EDTA exclusion experiments is unclear, but it suggests that any potential incremental release of NO• from Angeli's salt may be small.

### 4.1 Limitations

Angeli's salt was utilised as a source of HNO in this study, as it is the only commercially‐available HNO donor. As noted earlier, the co‐release of HNO and nitrite by Angeli's salt raises the possibility that the cardio‐ and vaso‐protective effects of Angeli's salt could be mediated, at least partially, by nitrite. The use of next‐generation HNO donors (e.g., BMS‐986231, cimlanod) that do not release active by‐products would eliminate this confounding factor (Velagic et al., 2020). However, these next‐generation HNO donors are currently undergoing clinical trials and are not yet commercially available (Kemp‐Harper et al., 2016). Other limitations are that we did not measure superoxide levels in hearts, as they were Langendorff‐perfused. To overcome this, future experiments could measure superoxide levels in the perfusate prior to construction of serial dose–response curves in Langendorff‐perfused hearts (Paolocci et al., 2001). The very acute nature of the exposure to HNO and NO• precluded determining their impact on sGC expression and activity. However, the mechanisms of action were explored via the well‐known pharmacological inhibitor, ODQ. Further, we did not incorporate assessment of basal coronary blood flow in vivo into our study design, hence whether there were any differences in basal coronary blood flow as a result of diabetes prior to the ex vivo interrogation of the dilator responses to Angeli's salt or DEA/NO was not determined. We also acknowledge that the rat model of T2DM used in this study did not exhibit obesity, which is a risk factor for T2DM and present in a large proportion of the human population with the disease (Almourani et al., 2019). Despite this, our animal model displayed other features of T2DM such as hyperglycaemia, hyperinsulinaemia, impaired glucose tolerance, decreased insulin sensitivity and cardiac insulin resistance. Diabetic rats also exhibited dyslipidaemia evident by higher plasma levels of triglycerides, lower HDL, and a higher total cholesterol to HDL ratio and LDL/VLDL to HDL ratio, relative to non‐diabetic rats. HDL levels are decreased in patients with T2DM and are an independent predictor of adverse cardiovascular events (Drexel et al., 2005; Grant & Meigs, 2007). Similarly, an increase in the total/HDL and LDL/HDL cholesterol ratios is predictive of greater cardiovascular risk (Millán et al., 2009). It should also be noted that performing a hyperinsulinaemic‐euglycaemic clamp would have improved the experimental design of this study, as this method is considered ‘gold standard’ in assessing insulin sensitivity in pre‐clinical models and humans with T2DM (Kim, 2009). A final limitation is that the precise contribution of hyperglycaemia, as distinct from diabetes per se, was not ascertained. In a previously reported clinical study, NO• resistance at the platelet level was directly proportional to extent of hyperglycaemia, as was superoxide production, and correction of hyperglycaemia ameliorated NO• resistance (Worthley et al., 2007). Similarly, Malmberg et al., showed that correction of hyperglycaemia improved long‐term prognosis in diabetic patients presenting with acute myocardial infarction: however, the contribution of amelioration of NO• resistance to improve prognosis was not evaluated (Malmberg et al., 1995).

## 5 CLINICAL IMPLICATIONS AND CONCLUSION

HNO donors have been clinically developed for heart failure‐related scenarios including heart failure with reduced ejection fraction (HFrEF) (Velagic et al., 2020) This is an arena where nitrates, which are the current standard therapy for acute heart failure, have not been able to enter, due both to their susceptibility to rapid development of nitrate tolerance (Irvine et al., 2011) and diminished efficacy in the presence of NO• resistance (Anderson et al., 2005). In comparison, repeated exposure to HNO donors does not lead to tolerance development (Irvine et al., 2011), and the efficacy of this drug class is maintained when responsiveness to NO• is impaired, creating a potential competitive advantage over nitrate therapy. This study reveals that HNO (released from Angeli's salt) circumvents impairments in vascular NO• signalling in a pre‐clinical model of T2DM by promoting vasorelaxation in the mesenteric and coronary vasculature. Further, the inotropic and lusitropic responses to HNO are preserved in the T2DM myocardium (in distinct contrast to NO•). These findings add to the growing evidence that HNO donors represent a new class of inotrope vasodilators that retain effectiveness in the context of diabetes‐induced vascular NO• resistance, further demonstrating the potential of HNO donors as a therapy for diabetes‐associated cardiovascular pathologies.

## AUTHOR CONTRIBUTIONS

C.X.Q., O.L.W., B.K.K‐H., and R.H.R. conceived and designed the research. C.X.Q., S.A.M., O.L.W., and B.K.K‐H. provided training for Langendorff and wire myography techniques. A.V., J.C.L, M.L., and M.D. conducted animal experiments and collected the data. A.V. performed wire myography, Langendorff, and western blotting experiments. D.A. performed mass spectrometry. A.V. analysed the data, prepared figures, and drafted the manuscript. A.V., C.X.Q., O.L.W., J.D.H., B.K.K‐H., and R.H.R. interpreted the data. A.V., C.X.Q., S.A.M., O.L.W., J.D.H., B.K.K‐H., and R.H.R. edited and revised the manuscript. All authors approved the final version of the manuscript.

## CONFLICT OF INTEREST

All authors have nothing to declare.

## DECLARATION OF TRANSPARENCY AND SCIENTIFIC RIGOUR

This Declaration acknowledges that this paper adheres to the principles for transparent reporting and scientific rigour of preclinical research as stated in the *BJP* guidelines for Natural Products Research, Design and Analysis, Immunoblotting and Immunochemistry, and Animal Experimentation, and as recommended by funding agencies, publishers and other organisations engaged with supporting research.

## Supporting information


**Figure S1.** CONSAERT flow diagram for reporting animal use and analysis in preclinical studies.
**Figure S2.** Representative trace of protocol for non‐diabetic and diabetic Langendorff‐perfused hearts. Dose–response curves to DEA/NO and Angeli's salt, showing effects on perfusion pressure, left ventricular (LV) pressure, heart rate (HR), LV + dP/dt (maximal rate of rise in LV pressure), LV‐dP/dt (maximal rate of fall in LV pressure) and coronary flow rate. Perfusion pressure was held constant using the STH pump controller prior to U46619 infusion or insulin (33.3 IU/mL, 0.1 mL) administration. There was a 10 minute washout period with Krebs buffer between dose–response curves and prior to insulin administration. Approximately 3 minutes after insulin was administered, left ventricles were isolated and snap frozen.
**Figure S3.** Representative trace of wire myography protocol in mesenteric arteries isolated from non‐diabetic and diabetic rats. Cumulative‐concentration response curve to Angeli's salt constructed following normalisation protocol, maximal contraction response to KPSS (100 mM) and assessment of endothelial integrity using acetylcholine (ACh, 10^−5^ M).
**Figure S4.** Comparison of metabolic characteristics between non‐diabetic and diabetic rats. (A) Fortnightly body weight and (B) blood glucose levels. (C) Glucose tolerance (D) and insulin tolerance test at 11 weeks of diabetes. Data are presented as mean ± SEM. Data analysed by two‐way repeated measures ANOVA with Sidak's post‐hoc test for multiple comparisons, or Student's unpaired t‐test, where appropriate. *P < 0.05 compared to the non‐diabetic group. STZ, streptozotocin; GTT, glucose tolerance test; ITT, insulin tolerance test; AUC, area under the curve.
**Figure S5.** Change in (A) LVSP, (B) LVEDP and (C) heart rate in response to insulin in Langendorff‐perfused hearts isolated from non‐diabetic or diabetic rats. Data are expressed as the change from baseline (denoted by ∆), mean ± SEM. Data analysed by Student's unpaired t‐test. *P < 0.05 compared to the non‐diabetic group. ∆, change from baseline; LV, left ventricular; LVSP, LV systolic pressure; LVEDP, LV end‐diastolic pressure.
**Figure S6.** LV protein expression of (A) phospho‐eNOS/total‐eNOS, (B) phospho‐eNOS/β‐actin and (C) total‐eNOS/β‐actin following a bolus dose of insulin in non‐diabetic and diabetic Langendorff‐perfused hearts. (D) Representative immunoblot of phospho‐eNOS, total‐eNOS and β‐actin. Uncropped blots are provided in Supplementary Figure 8. LV, left ventricular. eNOS, endothelial nitric oxide synthase.
**Figure S7.** Complete LV immunoblot of (A,B) phospho‐Akt, (C,D) total‐Akt and (E,F) β‐actin. Red boxes represent cropped areas included in representative immunoblots in Figure 1. ND, non‐diabetic; D, diabetic.
**Figure S8.** Complete LV immunoblot of (A,B) phospho‐eNOS, (C,D) total‐eNOS and (E,F) β‐actin. Red boxes represent cropped areas included in representative immunoblots in Supplementary Figure 6. ND, non‐diabetic; D, diabetic. eNOS, endothelial nitric oxide synthase.
**Figure S9.** Complete LV immunoblot of (A,B) p22^phox^ and (C,D) β‐actin. Red boxes represent cropped areas included in representative immunoblots in Figure 1. ND, non‐diabetic; D, diabetic.
**Figure S10.** Dose–response curves to NO*•* donor DEA/NO or HNO donor Angeli's salt in Langendorff‐perfused hearts. LVEDP in response to DEA/NO or Angeli's salt in (A) non‐diabetic and (B) diabetic hearts. Data are expressed as change from baseline (denoted by ∆), mean ± SEM. Data analysed by two‐way repeated measures ANOVA with Sidak's post‐hoc test for multiple comparisons. *P < 0.05 compared to the non‐diabetic group NO*•*, nitric oxide; HNO, nitroxyl; LV, left ventricular; LVEDP, LV end‐diastolic pressure.
**Figure S11.** Impact of ODQ on dose–response curves to DEA/NO or Angeli's salt in non‐diabetic and diabetic Langendorff‐perfused hearts. LVSP in response to DEA/NO in (A) non‐diabetic and (B) diabetic hearts, or Angeli's salt in (C) non‐diabetic and (D) diabetic hearts. LVEDP in response to DEA/NO in (E) non‐diabetic and (F) diabetic hearts, or Angeli's salt in (G) non‐diabetic and (H) diabetic hearts. Data are expressed as change from baseline (denoted by ∆), mean ± SEM. Data analysed by two‐way repeated measures ANOVA with Sidak's post‐hoc test for multiple comparisons. *P < 0.05 compared to absence of ODQ. NO*•*, nitric oxide; HNO, nitroxyl; LV, left ventricular; LVSP, LV systolic pressure; LVEDP, LV end‐diastolic pressure; ODQ, 1H‐[1,2,4]oxadiazolo[4,3‐a]quinoxaline‐1‐one (sGC inhibitor).
**Figure S12.** Cumulative concentration‐response curves to the HNO donor Angeli's salt in mesenteric arteries from non‐diabetic and diabetic rats in the absence or presence of either (A,B) the NO• scavenger HXC (100 μmol/L) or (C,D,E) the sGC inhibitor ODQ (10 μmol/L). Values are expressed as % reversal of pre‐contraction and given as mean ± SEM, *n* = number of animals. HNO, nitroxyl; NO•, nitric oxide; HXC, hydroxocobalamin; ODQ, 1H‐[1,2,4]oxadiazolo[4,3‐a]quinoxaline‐1‐one.
**Figure S13.** Schematic diagram summarising the impact of diabetes on responses to various agonists in the myocardium, and coronary and mesenteric vasculature. DEA/NO, nitric oxide donor; Angeli's salt, nitroxyl donor; ODQ, 1H‐[1,2,4]oxadiazolo[4,3‐a]quinoxaline‐1‐one (soluble guanylate cyclase inhibitor); ACh, acetylcholine (endothelium‐dependent vasodilator); SNP, sodium nitroprusside (endothelium‐independent vasodilator); HXC, hydroxocobalamin (nitric oxide scavenger); L‐cysteine, nitroxyl scavenger.
**Figure S14.** Cumulative concentration‐response curves to (A,B) DEA/NO, (C,D) Angeli's salt or (E,F) sodium nitrite in the presence or absence of EDTA. Responses were obtained in presence or absence of the NO• scavenger HXC (100 μmol/L) or the HNO scavenger L‐cysteine (3 mmol/L) in mesenteric arteries from control (untreated, naïve) rats. Values are expressed as % reversal of pre‐contraction and given as mean ± SEM, *n* = number of animals. *P < 0.05, pEC_50_ compared to control. HNO, nitroxyl; NO•, nitric oxide; HXC, hydroxocobalamin. pEC_50_, potency.
**Figure S15.** Metabolites of the methionine cycle, glutathione synthesis and the glutathione redox cycle. Methionine is converted to S‐adenosyl‐methionine (SAM) by methionine adenyosyltransferase (MAT). SAM is used as a methyl source by methyltransferases (MTs) and S‐adenosyl‐homocysteine (SAH) is formed as a by‐product. SAH is converted by adenosylhomocysteine (AHCY) to homocysteine, which is converted to methionine by methionine synthase (MS) or directed to the glutathione synthesis pathway (Sanderson et al., 2019). Cystathionine β‐synthase (CBS) catalyses the addition of homocysteine and serine to generate cystathionine, which is degraded to cysteine by cystathionine γ‐lyase (CSE). γ‐glutamyl cysteine synthetase (γ‐GCS) catalyses the combination of cysteine and glutamate to generate γ‐glutamylcysteine (γ‐Glu‐Cys). Glutathione synthetase (GSS) catalyses the condensation of γ‐Glu‐Cys and glycine to form glutathione (GSH) (Paul, 2021). GSH peroxidase reduces hydrogen peroxide (H_2_O_2_) and uses GSH as a co‐factor, resulting in the formation of oxidised glutathione (GSSG). GSH reductase reduces GSSG to GSH by using NADPH (Lubos et al., 2011). GSH exported into the extracellular space is converted to cysteinylglycine (Cys‐Gly) by γ‐glutamyltransferase (GGT). Cys‐Gly is converted to cysteine and glycine by dipeptidase (DP). In the extracellular space, cysteine is readily oxidised to cystine, which is imported into the cell for GSH synthesis (Banjac et al., 2008).
**Table S1.** Metabolic characteristics between non‐diabetic and diabetic rats at study end‐point (12 weeks post‐vehicle or ‐STZ).
**Table S2.** Haemodynamic parameters of non‐diabetic and diabetic Langendorff‐perfused hearts at baseline following equilibration, after pre‐treatment with ODQ, and during U46619 infusion prior to dose–response curves to DEA/NO or Angeli's salt.
**Table S3.** Relaxation to endothelium‐independent vasodilators in mesenteric arteries in the presence or absence of EDTA in Krebs' solution.

## Data Availability

The data that supports the findings of this study are also available from the corresponding authors upon reasonable request. Some data may not be available because of privacy or ethical restrictions.

## References

[bph15849-bib-0001] Alexander, S. P. H. , Kelly, E. , Mathie, A. , Peters, J. A. , Veale, E. L. , Armstrong, J. F. , Faccenda, E. , Harding, S. D. , Pawson, A. J. , Southan, C. , Buneman, O. P. , Cidlowski, J. A. , Christopoulos, A. , Davenport, A. P. , Fabbro, D. , Spedding, M. , Striessnig, J. , Davies, J. A. , Ahlers‐Dannen, K. E. , … Zolghadri, Y. (2021). The concise guide to pharmacology 2021/22: Introduction and other protein targets. British Journal of Pharmacology, 178(S1), S1–S26. 10.1111/bph.15537 34529830 PMC9513948

[bph15849-bib-0002] Alexander, S. P. H. , Roberts, R. E. , Broughton, B. R. S. , Sobey, C. G. , George, C. H. , Stanford, S. C. , Cirino, G. , Docherty, J. R. , Giembycz, M. A. , Hoyer, D. , Insel, P. A. , Izzo, A. A. , Ji, Y. , MacEwan, D. J. , Mangum, J. , Wonnacott, S. , & Ahluwalia, A. (2018). Goals and practicalities of immunoblotting and immunohistochemistry: A guide for submission to the *British Journal of Pharmacology* . British Journal of Pharmacology, 175(3), 407–411. 10.1111/bph.14112 29350411 PMC5773976

[bph15849-bib-0003] Almourani, R. , Chinnakotla, B. , Patel, R. , Kurukulasuriya, L. R. , & Sowers, J. (2019). Diabetes and cardiovascular disease: An update. Current Diabetes Reports, 19(12), 161. 10.1007/s11892-019-1239-x 31828525

[bph15849-bib-0004] Al‐Salameh, A. , Chanson, P. , Bucher, S. , Ringa, V. , & Becquemont, L. (2019). Cardiovascular disease in type 2 diabetes: A review of sex‐related differences in predisposition and prevention. Mayo Clinic Proceedings, 94(2), 287–308. 10.1016/j.mayocp.2018.08.007 30711127

[bph15849-bib-0005] Anderson, E. J. , Kypson, A. P. , Rodriguez, E. , Anderson, C. A. , Lehr, E. J. , & Neufer, D. P. (2009). Substrate‐specific derangements in mitochondrial metabolism and redox balance in the atrium of the type 2 diabetic human heart. Journal of the American College of Cardiology, 54(20), 1891–1898. 10.1016/j.jacc.2009.07.031 19892241 PMC2800130

[bph15849-bib-0006] Anderson, R. A. , Ellis, G. R. , Chirkov, Y. Y. , Holmes, A. S. , Payne, N. , Blackman, D. J. , Jackson, S. K. , Lewis, M. J. , Horowitz, J. D. , & Frenneaux, M. P. (2004). Determinants of platelet responsiveness to nitric oxide in patients with chronic heart failure. European Journal of Heart Failure, 6(1), 47–54. 10.1016/S1388-9842(03)00038-2 15012918

[bph15849-bib-0007] Anderson, R. A. , Ellis, G. R. , Evans, L. M. , Morris, K. , Chirkov, Y. Y. , Horowitz, J. D. , Jackson, S. K. , Rees, A. , Lewis, M. J. , & Frenneaux, M. P. (2005). Platelet nitrate responsiveness in fasting and postprandial type 2 diabetes. Diabetes & Vascular Disease Research, 2(2), 88–93. 10.3132/dvdr.2005.015 16305064

[bph15849-bib-0008] Aquilano, K. , Baldelli, S. , & Ciriolo, M. R. (2014). Glutathione: New roles in redox signalling for an old antioxidant. Frontiers in Pharmacology, 5, 196. 10.3389/fphar.2014.00196 25206336 PMC4144092

[bph15849-bib-0009] Chin, K. Y. , Qin, C. , Cao, N. , Kemp‐Harper, B. K. , Woodman, O. L. , & Ritchie, R. H. (2014). The concomitant coronary vasodilator and positive inotropic actions of the nitroxyl donor Angeli's salt in the intact rat heart: Contribution of soluble guanylyl cyclase‐dependent and ‐independent mechanisms. British Journal of Pharmacology, 171(7), 1722–1734. 10.1111/bph.12568 24372173 PMC3966751

[bph15849-bib-0010] Dautov, R. F. , Ngo, D. T. M. , Licari, G. , Liu, S. , Sverdlov, A. L. , Ritchie, R. H. , Kemp‐Harper, B. K. , Horowitz, J. D. , & Chirkov, Y. Y. (2013). The nitric oxide redox sibling nitroxyl partially circumvents impairment of platelet nitric oxide responsiveness. Nitric Oxide: Biology and Chemistry, 35, 72–78. 10.1016/j.niox.2013.08.006 24012721

[bph15849-bib-0011] Drexel, H. , Aczel, S. , Marte, T. , Benzer, W. , Langer, P. , Moll, W. , & Saely, C. H. (2005). Is atherosclerosis in diabetes and impaired fasting glucose driven by elevated LDL cholesterol or by decreased HDL cholesterol? Diabetes Care, 28(1), 101–107. 10.2337/diacare.28.1.101 15616241

[bph15849-bib-0012] Drucker, D. J. (2016). Never waste a good crisis: Confronting reproducibility in translational research. Cell Metabolism, 24(3), 348–360. 10.1016/j.cmet.2016.08.006 27626191

[bph15849-bib-0013] Drummond, G. R. , & Sobey, C. G. (2014). Endothelial NADPH oxidases: Which NOX to target in vascular disease? Trends in Endocrinology and Metabolism, 25(9), 452–463. 10.1016/j.tem.2014.06.012 25066192

[bph15849-bib-0014] El‐Seweidy, M. M. , Sadik, N. A. H. , & Shaker, O. G. (2011). Role of sulfurous mineral water and sodium hydrosulfide as potent inhibitors of fibrosis in the heart of diabetic rats. Archives of Biochemistry and Biophysics, 506(1), 48–57. 10.1016/j.abb.2010.10.014 20965145

[bph15849-bib-0015] Favaloro, J. L. , & Kemp‐Harper, B. K. (2007). The nitroxyl anion (HNO) is a potent dilator of rat coronary vasculature. Cardiovascular Research, 73(3), 587–596. 10.1016/j.cardiores.2006.11.018 17189622

[bph15849-bib-0016] Furman, B. L. (2015). Streptozotocin‐induced diabetic models in mice and rats. Current Protocols in Pharmacology, 70(1), 5–47. 10.1002/0471141755.ph0547s70 26331889

[bph15849-bib-0017] Grant, R. W. , & Meigs, J. B. (2007). Prevalence and treatment of low HDL cholesterol among primary care patients with type 2 diabetes: An unmet challenge for cardiovascular risk reduction. Diabetes Care, 30(3), 479–484. 10.2337/dc06-1961 17327308

[bph15849-bib-0018] Hamblin, M. , Friedman, D. B. , Hill, S. , Caprioli, R. M. , Smith, H. M. , & Hill, M. F. (2007). Alterations in the diabetic myocardial proteome coupled with increased myocardial oxidative stress underlies diabetic cardiomyopathy. Journal of Molecular and Cellular Cardiology, 42(4), 884–895. 10.1016/j.yjmcc.2006.12.018 17320100 PMC2677446

[bph15849-bib-0019] International Diabetes Federation . (2019). Latest figures show 463 million people now living with diabetes worldwide as numbers continue to rise. Diabetes Research and Clinical Practice, 157, 107932. 10.1016/j.diabres.2019.107932 31806126

[bph15849-bib-0020] Irvine, J. C. , Cao, N. , Gossain, S. , Alexander, A. E. , Love, J. E. , Qin, C. , Horowitz, J. D. , Kemp‐Harper, B. K. , & Ritchie, R. H. (2013). HNO/cGMP‐dependent antihypertrophic actions of isopropylamine‐NONOate in neonatal rat cardiomyocytes: Potential therapeutic advantages of HNO over NȮ. American Journal of Physiology ‐ Heart and Circulatory Physiology, 305(3), 365–377. 10.1152/ajpheart.00495.2012 23729209

[bph15849-bib-0021] Irvine, J. C. , Favaloro, J. L. , & Kemp‐Harper, B. K. (2003). NO‐ activates soluble guanylate cyclase and Kv channels to vasodilate resistance arteries. Hypertension, 41(6), 1301–1307. 10.1161/01.HYP.0000072010.54901.DE 12743008

[bph15849-bib-0022] Irvine, J. C. , Kemp‐Harper, B. K. , & Widdop, R. E. (2011). Chronic administration of the HNO donor Angeli's salt does not lead to tolerance, cross‐tolerance, or endothelial dysfunction: Comparison with GTN and DEA/NO. Antioxidants & Redox Signaling, 14(9), 1615–1624. 10.1089/ars.2010.3269 20849324

[bph15849-bib-0023] Izzo, A. A. , Teixeira, M. , Alexander, S. P. , Cirino, G. , Docherty, J. R. , George, C. H. , Insel, P. A. , Ji, Y. , Kendall, D. A. , Panattieri, R. A. , Sobey, C. G. , Stanford, S. C. , Stefanska, B. , Stephens, G. , & Ahluwalia, A. (2020). A practical guide for transparent reporting of research on natural products in the *British Journal of Pharmacology*: Reproducibility of natural product research. British Journal of Pharmacology, 177(10), 2169–2178. 10.1111/bph.15054 32298474 PMC7174877

[bph15849-bib-0024] Jagasia, D. , Whiting, J. M. , Concato, J. , Pfau, S. , & McNulty, P. H. (2001). Effect of non‐insulin‐dependent diabetes mellitus on myocardial insulin responsiveness in patients with ischemic heart disease. Circulation, 103(13), 1734–1739. 10.1161/01.CIR.103.13.1734 11282903

[bph15849-bib-0025] Keceli, G. , Majumdar, A. , Thorpe, C. N. , Jun, S. , Tocchetti, C. G. , Lee, D. I. , Mahaney, J. E. , Paolocci, N. , & Toscano, J. P. (2019). Nitroxyl (HNO) targets phospholamban cysteines 41 and 46 to enhance cardiac function. The Journal of General Physiology, 151(6), 758–770. 10.1085/jgp.201812208 30842219 PMC6571998

[bph15849-bib-0026] Kemp‐Harper, B. K. , Horowitz, J. D. , & Ritchie, R. H. (2016). Therapeutic potential of nitroxyl (HNO) donors in the management of acute decompensated heart failure. Drugs, 76(14), 1337–1348. 10.1007/s40265-016-0631-y 27566478

[bph15849-bib-0027] Kemp‐Harper, B. K. , & Schmidt, H. H. H. W. (2009). In H. H. H. W. Schmidt , F. Hofmann , & J.‐P. Stasch (Eds.), cGMP: Generators, effectors and therapeutic implications (pp. 447–467). Springer. 10.1007/978-3-540-68964-5_19 19192509

[bph15849-bib-0028] Kim, J. K. (2009). In C. Stocker (Ed.), Hyperinsulinemic–euglycemic clamp to assess insulin sensitivity in vivo BT—Type 2 diabetes: Methods and protocols (pp. 221–238). Humana Press. 10.1007/978-1-59745-448-3_15 19504253

[bph15849-bib-0029] Leo, C. H. , Hart, J. L. , & Woodman, O. L. (2011). Impairment of both nitric oxide‐mediated and EDHF‐type relaxation in small mesenteric arteries from rats with streptozotocin‐induced diabetes. British Journal of Pharmacology, 162(2), 365–377. 10.1111/j.1476-5381.2010.01023.x 20840539 PMC3031058

[bph15849-bib-0030] Leo, C. H. , Joshi, A. , Hart, J. L. , & Woodman, O. L. (2012). Endothelium‐dependent nitroxyl‐mediated relaxation is resistant to superoxide anion scavenging and preserved in diabetic rat aorta. Pharmacological Research, 66(5), 383–391. 10.1016/j.phrs.2012.07.010 22898326

[bph15849-bib-0031] Li, S. , Li, X. , Li, Y. L. , Shao, C. H. , Bidasee, K. R. , & Rozanski, G. J. (2007). Insulin regulation of glutathione and contractile phenotype in diabetic rat ventricular myocytes. American Journal of Physiology ‐ Heart and Circulatory Physiology, 292(3), 1619–1629. 10.1152/ajpheart.00140.2006 17056675

[bph15849-bib-0032] Lilley, E. , Stanford, S. C. , Kendall, D. E. , Alexander, S. P. H. , Cirino, G. , Docherty, J. R. , George, C. H. , Insel, P. A. , Izzo, A. A. , Ji, Y. , Panettieri, R. A. , Sobey, C. G. , Stefanska, B. , Stephens, G. , Teixeira, M. , & Ahluwalia, A. (2020). ARRIVE 2.0 and the *British Journal of Pharmacology*: Updated guidance for 2020. British Journal of Pharmacology, 177(16), 3611–3616. 10.1111/bph.15178 32662875 PMC7393193

[bph15849-bib-0033] Lin, E. Q. , Irvine, J. C. , Cao, A. H. , Alexander, A. E. , Love, J. E. , Patel, R. , McMullen, J. R. , Kaye, D. M. , Kemp‐Harper, B. K. , & Ritchie, R. H. (2012). Nitroxyl (HNO) stimulates soluble guanylyl cyclase to suppress cardiomyocyte hypertrophy and superoxide generation. PLoS ONE, 7(4), 6–16. 10.1371/journal.pone.0034892 PMC3323591 22506056

[bph15849-bib-0034] Maguire, S. M. , Nugent, A. G. , McGurk, C. , Johnston, G. D. , & Nicholls, D. P. (1998). Abnormal vascular responses in human chronic cardiac failure are both endothelium dependent and endothelium independent. Heart, 80(2), 141–145. 10.1136/hrt.80.2.141 9813559 PMC1728779

[bph15849-bib-0035] Maier, S. , Aulbach, F. , Simm, A. , Lange, V. , Langenfeld, H. , Behre, H. , Kersting, U. , Walter, U. , & Kirstein, M. (1999). Stimulation of L‐type Ca2+ current in human atrial myocytes by insulin. Cardiovascular Research, 44(2), 390–397. 10.1016/S0008-6363(99)00229-1 10690315

[bph15849-bib-0036] Malmberg, K. , Rydén, L. , Efendic, S. , Herlitz, J. , Nicol, P. , Waldenstrom, A. , Wedel, H. , & Welin, L. (1995). Randomized trial of insulin‐glucose infusion followed by subcutaneous insulin treatment in diabetic patients with acute myocardial infarction (DIGAMI study): Effects on mortality at 1 year. Journal of the American College of Cardiology, 26(1), 57–65. 10.1016/0735-1097(95)00126-K 7797776

[bph15849-bib-0037] Marsh, S. A. , Dell′Italia, L. J. , & Chatham, J. C. (2009). Interaction of diet and diabetes on cardiovascular function in rats. American Journal of Physiology. Heart and Circulatory Physiology, 296(2), H282–H292. 10.1152/ajpheart.00421.2008 19036853 PMC2643886

[bph15849-bib-0038] Millán, J. , Pintó, X. , Muñoz, A. , Zúñiga, M. , Rubiés‐Prat, J. , Pallardo, L. F. , Masana, L. , Mangas, A. , Hernández‐Mijares, A. , González‐Santos, P. , Ascaso, J. F. , & Pedro‐Botet, J. (2009). Lipoprotein ratios: Physiological significance and clinical usefulness in cardiovascular prevention. Vascular Health and Risk Management, 5, 757–765. https://pubmed.ncbi.nlm.nih.gov/19774217 19774217 PMC2747394

[bph15849-bib-0039] Miller, T. W. , Cherney, M. M. , Lee, A. J. , Francoleon, N. E. , Farmer, P. J. , King, S. B. , Hobbs, A. J. , Miranda, K. M. , Burstyn, J. N. , & Fukuto, J. M. (2009). The effects of nitroxyl (HNO) on soluble guanylate cyclase activity: Interactions at ferrous heme and cysteine thiols. The Journal of Biological Chemistry, 284(33), 21788–21796. 10.1074/jbc.M109.014282 19531488 PMC2755905

[bph15849-bib-0040] Miranda, K. M. , Nims, R. W. , Thomas, D. D. , Espey, M. G. , Citrin, D. , Bartberger, M. D. , Paolocci, N. , Fukuto, J. M. , Feelisch, M. , & Wink, D. A. (2003). Comparison of the reactivity of nitric oxide and nitroxyl with heme proteins: A chemical discussion of the differential biological effects of these redox related products of NOS. Journal of Inorganic Biochemistry, 93(1), 52–60. 10.1016/S0162-0134(02)00498-1 12538052

[bph15849-bib-0041] Muniyappa, R. , Montagnani, M. , Koh, K. K. , & Quon, M. J. (2007). Cardiovascular actions of insulin. Endocrine Reviews, 28(5), 463–491. 10.1210/er.2007-0006 17525361

[bph15849-bib-0042] Ohkuma, T. , Komorita, Y. , Peters, S. A. E. , & Woodward, M. (2019). Diabetes as a risk factor for heart failure in women and men: A systematic review and meta‐analysis of 47 cohorts including 12 million individuals. Diabetologia, 62(9), 1550–1560. 10.1007/s00125-019-4926-x 31317230 PMC6677875

[bph15849-bib-0043] Ortmayr, K. , Schwaiger, M. , Hann, S. , & Koellensperger, G. (2015). An integrated metabolomics workflow for the quantification of sulfur pathway intermediates employing thiol protection with N‐ethyl maleimide and hydrophilic interaction liquid chromatography tandem mass spectrometry. Analyst, 140(22), 7687–7695. 10.1039/c5an01629k 26451393

[bph15849-bib-0044] Pacher, P. , Beckman, J. S. , & Liaudet, L. (2007). Nitric oxide and peroxynitrite in health and disease. Physiological Reviews, 87(1), 315–424. 10.1152/physrev.00029.2006 17237348 PMC2248324

[bph15849-bib-0045] Paolocci, N. , Biondi, R. , Bettini, M. , Lee, C. I. , Berlowitz, C. O. , Rossi, R. , Xia, Y. , Ambrosio, G. , L'Abbate, A. , Kass, D. A. , & Zweier, J. L. (2001). Oxygen radical‐mediated reduction in basal and agonist‐evoked no release in isolated rat heart. Journal of Molecular and Cellular Cardiology, 33(4), 671–679. 10.1006/jmcc.2000.1334 11341236

[bph15849-bib-0046] Paulus, W. J. , & Bronzwaer, J. G. F. (2004). Nitric oxide's role in the heart: Control of beating or breathing? American Journal of Physiology ‐ Heart and Circulatory Physiology, 287(1), H8–H13. 10.1152/ajpheart.01147.2003 15210448

[bph15849-bib-0047] Percie du Sert, N. , Hurst, V. , Ahluwalia, A. , Alam, S. , Avey, M. T. , Baker, M. , Browne, W. J. , Clark, A. , Cuthill, I. C. , Dirnagl, U. , Emerson, M. , Garner, P. , Holgate, S. T. , Howells, D. W. , Karp, N. A. , Lazic, S. E. , Lidster, K. , MacCallum, C. J. , Macleod, M. , … Würbel, H. (2020). The ARRIVE guidelines 2.0: Updated guidelines for reporting animal research. PLoS Biology, 18(7), e3000410. 10.1371/journal.pbio.3000410 32663219 PMC7360023

[bph15849-bib-0048] Qin, C. X. , Anthonisz, J. , Leo, C. H. , Kahlberg, N. , Velagic, A. , Li, M. , Jap, E. , Woodman, O. L. , Parry, L. J. , Horowitz, J. D. , Kemp‐Harper, B. K. , & Ritchie, R. H. (2020). Nitric oxide resistance, induced in the myocardium by diabetes, is circumvented by the nitric oxide redox sibling, nitroxyl. Antioxidants & Redox Signaling, 32(1), 60–77. 10.1089/ars.2018.7706 31680536

[bph15849-bib-0049] Sasso, F. C. , Carbonara, O. , Cozzolino, D. , Rambaldi, P. , Mansi, L. , Torella, D. , Gentile, S. , Turco, S. , Torella, R. , & Salvatore, T. (2000). Effects of insulin‐glucose infusion of left ventricular function at rest and during dynamic exercise in healthy subjects and noninsulin dependent diabetic patients. Journal of the American College of Cardiology, 36(1), 219–226. 10.1016/S0735-1097(00)00717-8 10898438

[bph15849-bib-0050] Schachinger, V. , Britten, M. B. , & Zeiher, A. M. (2000). Prognostic impact of coronary vasodilator dysfunction on adverse long‐term outcome of coronary heart disease. Circulation, 101(16), 1899–1906. 10.1161/01.CIR.101.16.1899 10779454

[bph15849-bib-0051] Shah, K. S. , Xu, H. , Matsouaka, R. A. , Bhatt, D. L. , Heidenreich, P. A. , Hernandez, A. F. , Devore, A. D. , Yancy, C. W. , & Fonarow, G. C. (2017). Heart failure with preserved, borderline, and reduced ejection fraction: 5‐year outcomes. Journal of the American College of Cardiology, 70(20), 2476–2486. 10.1016/j.jacc.2017.08.074 29141781

[bph15849-bib-0052] Tare, M. , Kalidindi, R. S. R. , Bubb, K. J. , Parkington, H. C. , Boon, W. M. , Li, X. , Sobey, C. G. , Drummond, G. R. , Ritchie, R. H. , & Kemp‐Harper, B. K. (2017). Vasoactive actions of nitroxyl (HNO) are preserved in resistance arteries in diabetes. Naunyn‐Schmiedeberg's Archives of Pharmacology, 390(4), 397–408. 10.1007/s00210-016-1336-1 28074232

[bph15849-bib-0053] Tate, M. , Prakoso, D. , Willis, A. M. , Peng, C. , Deo, M. , Qin, C. X. , Walsh, J. L. , Nash, D. M. , Cohen, C. D. , Rofe, A. K. , Sharma, A. , Kiriazis, H. , Donner, D. G. , De Haan, J. B. , Watson, A. M. D. , De Blasio, M. J. , & Ritchie, R. H. (2019). Characterising an alternative murine model of diabetic cardiomyopathy. Frontiers in Physiology, 10, 1–15. 10.3389/fphys.2019.01395 31798462 PMC6868003

[bph15849-bib-0054] Tocchetti, C. G. , Wang, W. , Froehlich, J. P. , Huke, S. , Aon, M. A. , Wilson, G. M. , di Benedetto, G. , O'Rourke, B. , Gao, W. D. , Wink, D. A. , Toscano, J. P. , Zaccolo, M. , Bers, D. M. , Valdivia, H. H. , Cheng, H. , Kass, D. A. , & Paolocci, N. (2007). Nitroxyl improves cellular heart function by directly enhancing cardiac sarcoplasmic reticulum Ca2+ cycling. Circulation Research, 100(1), 96–104. 10.1161/01.RES.0000253904.53601.c9 17138943 PMC2769513

[bph15849-bib-0055] van Etten, R. , de Koning, E. , Verhaar, M. , Gaillard, C. , & Rabelink, T. (2002). Impaired NO‐dependent vasodilation in patients with Type II (non‐insulin‐dependent) diabetes mellitus is restored by acute administration of folate. Diabetologia, 45(7), 1004–1010. 10.1007/s00125-002-0862-1 12136399

[bph15849-bib-0056] Velagic, A. , Qin, C. , Woodman, O. L. , Horowitz, J. D. , Ritchie, R. H. , & Kemp‐Harper, B. K. (2020). Nitroxyl: A novel strategy to circumvent diabetes associated impairments in nitric oxide signaling. Frontiers in Pharmacology, 11, 1–18. 10.3389/fphar.2020.00727 32508651 PMC7248192

[bph15849-bib-0057] Von Lewinski, D. , Bruns, S. , Walther, S. , Kögler, H. , & Pieske, B. (2005). Insulin causes [Ca2+]i‐dependent and [Ca 2+]i‐independent positive inotropic effects in failing human myocardium. Circulation, 111(20), 2588–2595. 10.1161/CIRCULATIONAHA.104.497461 15883206

[bph15849-bib-0058] Westerhof, N. , Boer, C. , Lamberts, R. R. , & Sipkema, P. (2006). Cross‐talk between cardiac muscle and coronary vasculature. Physiological Reviews, 86(4), 1263–1308. 10.1152/physrev.00029.2005 17015490

[bph15849-bib-0059] Wigg, S. J. , Tare, M. , Tonta, M. A. , O'Brien, R. C. , Meredith, I. T. , & Parkington, H. C. (2001). Comparison of effects of diabetes mellitus on an EDHF‐dependent and an EDHF‐independent artery. American Journal of Physiology ‐ Heart and Circulatory Physiology, 281(1), H232–H240. 10.1152/ajpheart.2001.281.1.h232 11406490

[bph15849-bib-0060] Williams, R. , Karuranga, S. , Malanda, B. , Saeedi, P. , Basit, A. , Besançon, S. , Bommer, C. , Esteghamati, A. , Ogurtsova, K. , Zhang, P. , & Colagiuri, S. (2020). Global and regional estimates and projections of diabetes‐related health expenditure: Results from the International Diabetes Federation Diabetes Atlas, 9th edition. Diabetes Research and Clinical Practice, 162, 108072. 10.1016/j.diabres.2020.108072 32061820

[bph15849-bib-0061] Williams, S. B. , Cusco, J. A. , Roddy, M. A. , Johnstone, M. T. , & Creager, M. A. (1996). Impaired nitric oxide‐mediated vasodilation in patients with non‐insulin‐dependent diabetes mellitus. Journal of the American College of Cardiology, 27(3), 567–574. 10.1016/0735-1097(95)00522-6 8606266

[bph15849-bib-0062] Worthley, M. I. , Holmes, A. S. , Willoughby, S. R. , Kucia, A. M. , Heresztyn, T. , Stewart, S. , Chirkov, Y. Y. , Zeitz, C. J. , & Horowitz, J. D. (2007). The deleterious effects of hyperglycemia on platelet function in diabetic patients with acute coronary syndromes: Mediation by superoxide production, resolution with intensive insulin administration. Journal of the American College of Cardiology, 49(3), 304–310. 10.1016/j.jacc.2006.08.053 17239711

[bph15849-bib-0063] Xu, Z. , Patel, K. P. , Lou, M. F. , & Rozanski, G. J. (2002). Up‐regulation of K(+) channels in diabetic rat ventricular myocytes by insulin and glutathione. Cardiovascular Research, 53(1), 80–88. 10.1016/S0008-6363(01)00446-1 11744015

[bph15849-bib-0064] Zaccardi, F. , Webb, D. R. , Yates, T. , & Davies, M. J. (2016). Pathophysiology of type 1 and type 2 diabetes mellitus: A 90‐year perspective. Postgraduate Medical Journal, 92(1084), 63–69. 10.1136/postgradmedj-2015-133281 26621825

[bph15849-bib-0065] Zeller, A. , Wenzl, M. V. , Beretta, M. , Stessel, H. , Russwurm, M. , Koesling, D. , Schmidt, K. , & Mayer, B. (2009). Mechanisms underlying activation of soluble guanylate cyclase by the nitroxyl donor Angeli's salt. Molecular Pharmacology, 76(5), 1115–1122. 10.1124/mol.109.059915 19720727

[bph15849-bib-0066] Zhao, Y. , Brandish, P. E. , DiValentin, M. , Schelvis, J. P. M. , Babcock, G. T. , & Marletta, M. A. (2000). Inhibition of soluble guanylate cyclase by ODQ. Biochemistry, 39(35), 10848–10854. 10.1021/bi9929296 10978171

[bph15849-bib-0067] Zhou, X. , Ma, L. , Habibi, J. , Whaley‐Connell, A. , Hayden, M. R. , Tilmon, R. D. , Brown, A. N. , Kim, J. A. , DeMarco, V. G. , & Sowers, J. R. (2010). Nebivolol improves diastolic dysfunction and myocardial remodeling through reductions in oxidative stress in the zucker obese rat. Hypertension, 55(4), 880–888. 10.1161/HYPERTENSIONAHA.109.145136 20176997 PMC2841702

