# Supplementary material for: Cardioprotective actions of nitroxyl donor Angeli's salt are preserved in the diabetic heart and vasculature in the face of nitric oxide resistance
Source: Br J Pharmacol. 2022 Apr 26;179(16):4117–35. doi: 10.1111/bph.15849 (PMC9540873; doi:10.1111/bph.15849)
Supplement: Supplementary file 1 — Figure S1. CONSAERT flow diagram for reporting animal use and analysis in preclinical studies. Figure S2. Representative trace of protocol for non‐diabetic and diabetic Langendorff‐perfused hearts. Dose–response curves to DEA/NO and Angeli's salt, showing effects on perfusion pressure, left ventricular (LV) pressure, heart rate (HR), LV + dP/dt (maximal rate of rise in LV pressure), LV‐dP/dt (maximal rate of fall in LV pressure) and coronary flow rate. Perfusion pressure was held constant using the STH pump controller prior to U46619 infusion or insulin (33.3 IU/mL, 0.1 mL) administration. There was a 10 minute washout period with Krebs buffer between dose–response curves and prior to insulin administration. Approximately 3 minutes after insulin was administered, left ventricles were isolated and snap frozen. Figure S3. Representative trace of wire myography protocol in mesenteric arteries isolated from non‐diabetic and diabetic rats. Cumulative‐concentration response curve to Angeli's salt constructed following normalisation protocol, maximal contraction response to KPSS (100 mM) and assessment of endothelial integrity using acetylcholine (ACh, 10−5 M). Figure S4. Comparison of metabolic characteristics between non‐diabetic and diabetic rats. (A) Fortnightly body weight and (B) blood glucose levels. (C) Glucose tolerance (D) and insulin tolerance test at 11 weeks of diabetes. Data are presented as mean ± SEM. Data analysed by two‐way repeated measures ANOVA with Sidak's post‐hoc test for multiple comparisons, or Student's unpaired t‐test, where appropriate. *P < 0.05 compared to the non‐diabetic group. STZ, streptozotocin; GTT, glucose tolerance test; ITT, insulin tolerance test; AUC, area under the curve. Figure S5. Change in (A) LVSP, (B) LVEDP and (C) heart rate in response to insulin in Langendorff‐perfused hearts isolated from non‐diabetic or diabetic rats. Data are expressed as the change from baseline (denoted by ∆), mean ± SEM. Data analysed by Student's un [file BPH-179-4117-s001.docx]

**SUPPLEMENTARY INFORMATION**

**Cardioprotective Actions of Nitroxyl Donor Angeli’s Salt are Preserved in the Diabetic Heart and Vasculature in the Face of Nitric Oxide Resistance**

Anida Velagic^1,2^, Jasmin Chendi Li^1,2^, Cheng Xue Qin^1,2^, Mandy Li^1,3^, Minh Deo^1,2^, Sarah A. Marshall^4^, Dovile Anderson^2^, Owen L. Woodman^2^, John D. Horowitz^5^, Barbara K. Kemp-Harper^3^ and Rebecca H. Ritchie^1,2,3*^

^1^Baker Heart and Diabetes Institute, Melbourne, VIC, Australia

^2^Monash Institute of Pharmaceutical Sciences, Monash University, Melbourne, VIC, Australia

^3^Biomedicine Discovery Institute, Department of Pharmacology, Monash University, Melbourne, VIC, Australia

^4^The Ritchie Centre, Department of Obstetrics and Gynaecology, School of Clinical Sciences, Monash University, VIC, Australia

^5^Basil Hetzel Institute, Queen Elizabeth Hospital, University of Adelaide, SA, Australia

***Correspondence:**

Professor Rebecca H. Ritchie

Theme Leader, Drug Discovery Biology

Monash Institute of Pharmaceutical Sciences

Monash University (Parkville Campus)

381 Royal Parade, Parkville

Victoria, Australia 3052

Tel: + 61 3 99039319

Email: Rebecca.Ritchie@monash.edu

**Keywords:** nitroxyl, HNO, nitric oxide resistance, diabetes, type 2 diabetes, cardiovascular disease

**Figure S1.** CONSAERT flow diagram for reporting animal use and analysis in preclinical studies.

**Figure S2.** Representative trace of protocol for non-diabetic and diabetic Langendorff-perfused hearts. Dose-response curves to DEA/NO and Angeli’s salt, showing effects on perfusion pressure, left ventricular (LV) pressure, heart rate (HR), LV +dP/dt (maximal rate of rise in LV pressure), LV-dP/dt (maximal rate of fall in LV pressure) and coronary flow rate. Perfusion pressure was held constant using the STH pump controller prior to U46619 infusion or insulin (33.3 IU/mL, 0.1 mL) administration. There was a 10 minute washout period with Krebs buffer between dose-response curves and prior to insulin administration. Approximately 3 minutes after insulin was administered, left ventricles were isolated and snap frozen.


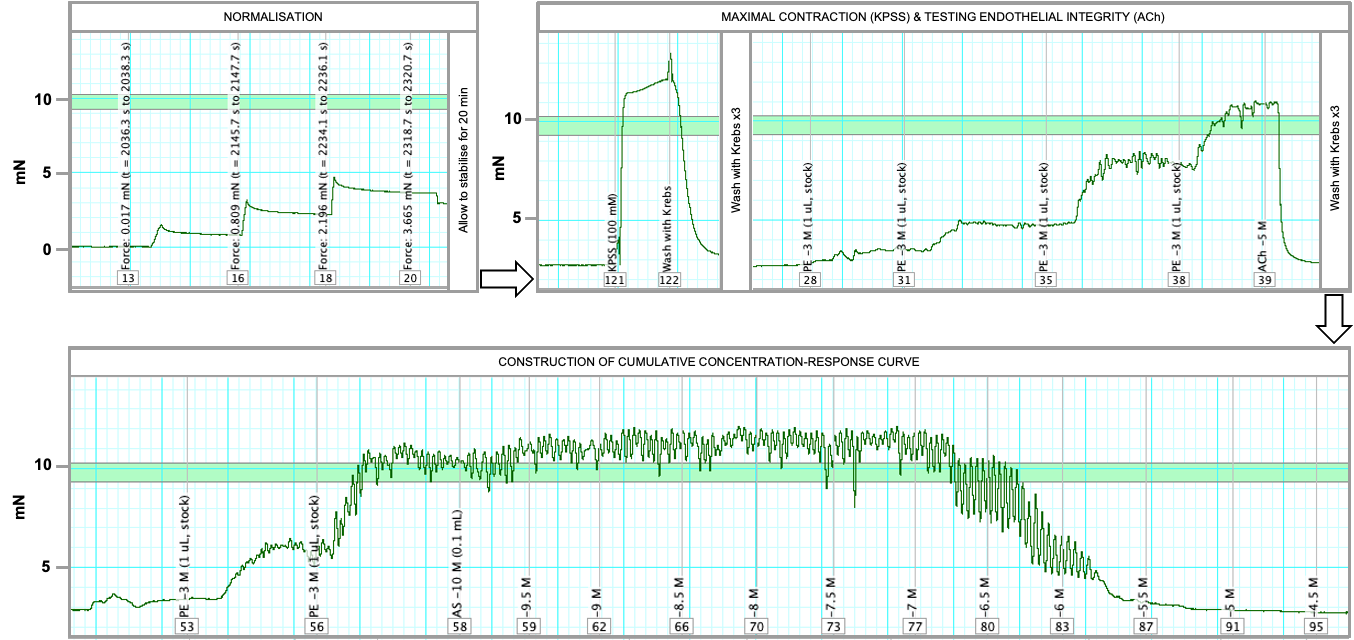
**Figure S3.** Representative trace of wire myography protocol in mesenteric arteries isolated from non-diabetic and diabetic rats. Cumulative-concentration response curve to Angeli’s salt constructed following normalisation protocol, maximal contraction response to KPSS (100 mM) and assessment of endothelial integrity using acetylcholine (ACh, 10^-5^ M).

**Figure S4.** Comparison of metabolic characteristics between non-diabetic and diabetic rats. (A) Fortnightly body weight and (B) blood glucose levels. (C) Glucose tolerance (D) and insulin tolerance test at 11 weeks of diabetes. Data are presented as mean ± SEM. Data analysed by two-way repeated measures ANOVA with Sidak’s post-hoc test for multiple comparisons, or Student’s unpaired t-test, where appropriate. **P<*0.05 compared to the non-diabetic group. STZ, streptozotocin; GTT, glucose tolerance test; ITT, insulin tolerance test; AUC, area under the curve.****

**Table S1.** Metabolic characteristics between non-diabetic and diabetic rats at study end-point (12 weeks post-vehicle or -STZ).

|  |  | Non-diabetic |  | Diabetic |
| --- | --- | --- | --- | --- |
|  | *n* |  | *n* |  |
| Body weight (g) |  | 614.2 ± 17.88 |  | 520.8 ± 18.07* |
| Whole blood |  |  |  |  |
| Blood glucose (mmol/L) | 20 | 6.63 ± 0.20 | 21 | 26.84 ± 0.99* |
| HbA1c (mmol/mol) | 20 | ND | 21 | 34.48 ± 1.65 |
| HbA1c (%) | 20 | ND | 21 | 5.31 ± 0.15 |
| Plasma |  |  |  |  |
| Insulin (ng/mL) | 8 | 0.49 ± 0.03 | 9 | 1.22 ± 0.28* |
| Triglyceride (mM) | 8 | 0.87 ± 0.24 | 9 | 3.02 ± 0.72* |
| Total cholesterol (mg/dL) | 7 | 49.84 ± 5.50 | 7 | 71.60 ± 9.64 |
| HDL (mg/dL) | 7 | 16.46 ± 2.60 | 7 | 9.86 ± 1.19* |
| LDL/VLDL (mg/dL) | 7 | 18.10 ± 2.91 | 7 | 25.88 ± 3.69 |
| Total cholesterol to HDL ratio | 7 | 3.31 ± 0.43 | 7 | 8.36 ± 1.75* |
| LDL/VLDL to HDL ratio | 7 | 1.13 ± 0.12 | 7 | 3.05 ± 0.67* |

Values are expressed as mean±SEM. *n* = number of animals. Data analysed by Student’s unpaired t-test. **P*<0.05 compared to the non-diabetic group. HbA1c, glycated haemoglobin; HDL, high-density lipoprotein; LDL/VLDL, low-density lipoprotein/very low-density lipoprotein. HbA1c in the non-diabetic group was below the detectable limit, and thus not detected (ND).

**Table S2.** Haemodynamic parameters of non-diabetic and diabetic Langendorff-perfused hearts at baseline following equilibration, after pre-treatment with ODQ, and during U46619 infusion prior to dose-response curves to DEA/NO or Angeli’s salt.

|  | **Non-diabetic** | | | **Non-diabetic + ODQ** | | | | **Diabetic** | | | **Diabetic + ODQ** | | | | |
| --- | --- | --- | --- | --- | --- | --- | --- | --- | --- | --- | --- | --- | --- | --- | --- |
|  | **Baseline** | **U46619 DEA/NO** | **U46619**  **AS** | **Baseline** | **ODQ** | **U46619**  **DEA/NO** | **U46619**  **AS** | **Baseline** | **U46619**  **DEA/NO** | **U46619**  **AS** | **Baseline** | **ODQ** | **U46619**  **DEA/NO** | **U46619**  **AS** |  |
| ***n*** | 8 | 8 | 8 | 7 | 7 | 7 | 7 | 8 | 8 | 9 | 9 | 9 | 9 | 9 |  |
| **Coronary flow (mL/min)** | 10.1±0.0 | 5.1±0.1 | 4.9±0.1 | 10.1±0.0 | 10.1±0.0 | 4.7±0.1 | 4.9±0.2 | 10.1±0.0 | 4.9±0.1 | 4.8±0.1 | 10.2±0.0 | 10.1±0.0 | 4.9±0.1 | 4.9±0.1 |  |
| **Perfusion pressure (mmHg)** | 56.8±6.0 | 55.4±1.9 | 55.0±1.7 | 50.4±2.6 | 51.3±3.0 | 51.9±2.3 | 51.4±2.6 | 58.7±7.0 | 58.1±4.4 | 55.2±3.4 | 51.5±1.9 | 52.2±1.5 | 50.9±1.3 | 49.7±1.3 |  |
| **LVSP (mmHg)** | 34.1±3.1 | 14.7±1.0 | 15.9±0.8 | 36.4±1.4 | 34.6±3.7 | 15.7±2.0 | 14.5±2.2 | 36.1±4.8 | 17.3±1.5 | 17.3±2.1 | 35.3±1.9 | 32.3±2.2 | 15.2±3.0 | 15.4±2.0 |  |
| **LVDP (mmHg)** | 45.8±3.8 | 25.8±2.7 | 27.7±2.4 | 42.1±2.5 | 44.1±4.6 | 26.2±3.0 | 20.7±3.3 | 43.5±4.9 | 24.4±2.5 | 23.3±2.9 | 41.0±3.0 | 37.7±3.3 | 22.7±2.9 | 20.2±2.3 |  |
| **LV+dP/dt (mmHg/sec)** | 1006±54 | 749±31 | 745±33 | 1053±49 | 1044±58 | 722±26 | 736±30 | 1048±50 | 771±40 | 774±36 | 1058±35 | 1044±48 | 757±26 | 710±35 |  |
| **LV-dP/dt (mmHg/sec)** | -898±47 | -629±45 | -664±46 | -873±30 | -832±24 | -621±27 | -628±26 | -915±56 | -638±45 | -651±55 | -867±25 | -825±21 | -624±26 | -613±25 |  |
| **Heart rate (beats/min)** | 206±5 | 205±4 | 208±9 | 203±5 | 200±3 | 205±5 | 204±4 | 162±7* | 171±8* | 174±8* | 173±6^#^ | 165±8^#^ | 175±8^#^ | 175±7^#^ |  |

Values are expressed as mean±SEM. *n* = number of animals. Data analysed by Student’s unpaired t-test. **P*<0.05 compared to the non-diabetic group. ^#^*P*<0.05 compared to the non-diabetic + ODQ group.

**Figure S5.** Change in (A) LVSP, (B) LVEDP and (C) heart rate in response to insulin in Langendorff-perfused hearts isolated from non-diabetic or diabetic rats. Data are expressed as the change from baseline (denoted by ∆), mean ± SEM. Data analysed by Student’s unpaired t-test. **P<*0.05 compared to the non-diabetic group. ∆, change from baseline; LV, left ventricular; LVSP, LV systolic pressure; LVEDP, LV end-diastolic pressure.

**Figure S6.** LV protein expression of (A) phospho-eNOS/total-eNOS, (B) phospho-eNOS/β-actin and (C) total-eNOS/β-actin following a bolus dose of insulin in non-diabetic and diabetic Langendorff-perfused hearts. (D) Representative immunoblot of phospho-eNOS, total-eNOS and β-actin. Uncropped blots are provided in Figure S8. LV, left ventricular. eNOS, endothelial nitric oxide synthase.

**Figure S7.** Complete LV immunoblot of (A,B) phospho-Akt, (C,D) total-Akt and (E,F) β-actin. Red boxes represent cropped areas included in representative immunoblots in Figure 1. ND, non-diabetic; D, diabetic.

**Figure S8.** Complete LV immunoblot of (A,B) phospho-eNOS, (C,D) total-eNOS and (E,F) β-actin. Red boxes represent cropped areas included in representative immunoblots in Figure S6. ND, non-diabetic; D, diabetic. eNOS, endothelial nitric oxide synthase.

**Figure S9.** Complete LV immunoblot of (A,B) p22^phox^ and (C,D) β-actin. Red boxes represent cropped areas included in representative immunoblots in Figure 1. ND, non-diabetic; D, diabetic.

**Figure S10.** Dose-response curves to NO*•* donor DEA/NO or HNO donor Angeli’s salt in Langendorff-perfused hearts. LVEDP in response to DEA/NO or Angeli’s salt in (A) non-diabetic and (B) diabetic hearts. Data are expressed as change from baseline (denoted by ∆), mean ± SEM. Data analysed by two-way repeated measures ANOVA with Sidak’s post-hoc test for multiple comparisons. **P<*0.05 compared to the non-diabetic group NO*•*, nitric oxide; HNO, nitroxyl; LV, left ventricular; LVEDP, LV end-diastolic pressure.

**Figure S11.** Impact of ODQ on dose-response curves to DEA/NO or Angeli’s salt in non-diabetic and diabetic Langendorff-perfused hearts. LVSP in response to DEA/NO in (A) non-diabetic and (B) diabetic hearts, or Angeli’s salt in (C) non-diabetic and (D) diabetic hearts. LVEDP in response to DEA/NO in (E) non-diabetic and (F) diabetic hearts, or Angeli’s salt in (G) non-diabetic and (H) diabetic hearts. Data are expressed as change from baseline (denoted by ∆), mean ± SEM. Data analysed by two-way repeated measures ANOVA with Sidak’s post-hoc test for multiple comparisons. **P<*0.05 compared to absence of ODQ. NO*•*, nitric oxide; HNO, nitroxyl; LV, left ventricular; LVSP, LV systolic pressure; LVEDP, LV end-diastolic pressure; ODQ, 1H-[1,2,4]oxadiazolo[4,3-a]quinoxaline-1-one (sGC inhibitor).

**Figure S12.** Cumulative concentration-response curves to the HNO donor Angeli’s salt in mesenteric arteries from non-diabetic and diabetic rats in the absence or presence of either (A,B) the NO• scavenger HXC (100 µmol/L) or (C,D,E) the sGC inhibitor ODQ (10 µmol/L). Values are expressed as % reversal of pre-contraction and given as mean ± SEM, *n* = number of animals. HNO, nitroxyl; NO•, nitric oxide; HXC, hydroxocobalamin; ODQ, 1H-[1,2,4]oxadiazolo[4,3-a]quinoxaline-1-one.


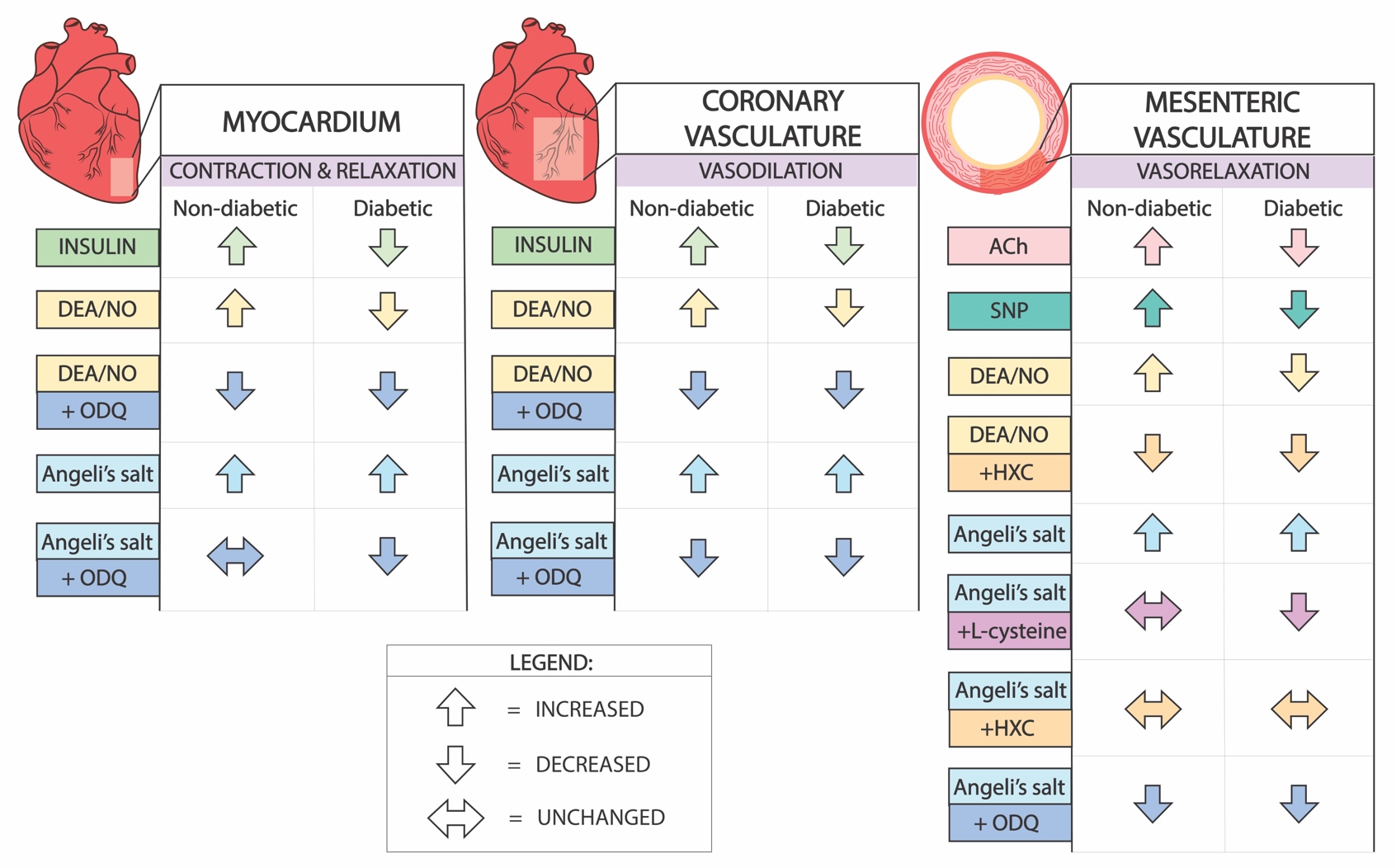
**Figure S13.** Schematic diagram summarising the impact of diabetes on responses to various agonists in the myocardium, and coronary and mesenteric vasculature. DEA/NO, nitric oxide donor; Angeli’s salt, nitroxyl donor; ODQ, 1H-[1,2,4]oxadiazolo[4,3-a]quinoxaline-1-one (soluble guanylate cyclase inhibitor); ACh, acetylcholine (endothelium-dependent vasodilator); SNP, sodium nitroprusside (endothelium-independent vasodilator); HXC, hydroxocobalamin (nitric oxide scavenger); L-cysteine, nitroxyl scavenger.

**Figure S14.** Cumulative concentration-response curves to (A,B) DEA/NO, (C,D) Angeli’s salt or (E,F) sodium nitrite in the presence or absence of EDTA. Responses were obtained in presence or absence of the NO• scavenger HXC (100 µmol/L) or the HNO scavenger L-cysteine (3 mmol/L) in mesenteric arteries from control (untreated, naïve) rats. Values are expressed as % reversal of pre-contraction and given as mean ± SEM, *n* = number of animals. **P*<0.05, pEC_50_ compared to control. HNO, nitroxyl; NO•, nitric oxide; HXC, hydroxocobalamin. pEC_50_, potency.

**Table S3.** Relaxation to endothelium-independent vasodilators in mesenteric arteries in the presence or absence of EDTA in Krebs’ solution.

|  | Control  Krebs’ solution with EDTA | | | Control  Krebs’ solution without EDTA | | |
| --- | --- | --- | --- | --- | --- | --- |
|  | *n* | pEC_50_ | R_max_ | *n* | pEC_50_ | R_max_ |
| DEA/NO | 5 | 6.95 ± 0.02 | 98 ± 1 | 5 | 7.17 ± 0.12 | 100 ± 0 |
| + HXC | 5 | 6.18 ± 0.09**^a^** | 98 ± 0 | 5 | 6.13 ± 0.12**^a^** | 98 ± 1 |
| + L-cysteine | 5 | 6.85 ± 0.17 | 100 ± 0 | 5 | 6.92 ± 0.07 | 100 ± 1 |
| Angeli’s salt | 5 | 6.28 ± 0.08 | 96 ± 1 | 5 | 6.37 ± 0.22 | 99 ± 0 |
| + HXC | 5 | 6.42 ± 0.10 | 96 ± 1 | 5 | 6.48 ± 0.03 | 99 ± 0 |
| + L-cysteine | 5 | 5.30 ± 0.12**^b^** | 92 ± 2 | 5 | 6.22 ± 0.15 | 98 ± 1 |
| Sodium nitrite | 5 | 5.13 ± 0.06**^c^** | 42 ± 5 | 5 | 5.33 ± 0.11**^c^** | 40 ± 3 |
| + HXC | 4 | 5.18 ± 0.13 | 40 ± 3 | 4 | 5.34 ± 0.13 | 41 ± 3 |
| + L-cysteine | 5 | 5.23 ± 0.09 | 43 ± 2 | 5 | 5.48 ± 0.11 | 39 ± 2 |

Values are expressed as mean ± SEM. pEC_50_ values are expressed as -log M. R_max_ values are expressed as % reversal of pre-contraction. *n* = number of animals. Data analysed by Student’s unpaired t-test. **^a^***P*<0.05 compared to DEA/NO. **^b^***P*<0.05 compared to Angeli’s salt. **^c^***P*<0.05 compared to Angeli’s salt. pEC_50_, potency; R_max_, maximum relaxation; HXC, hydroxocobalamin.

**
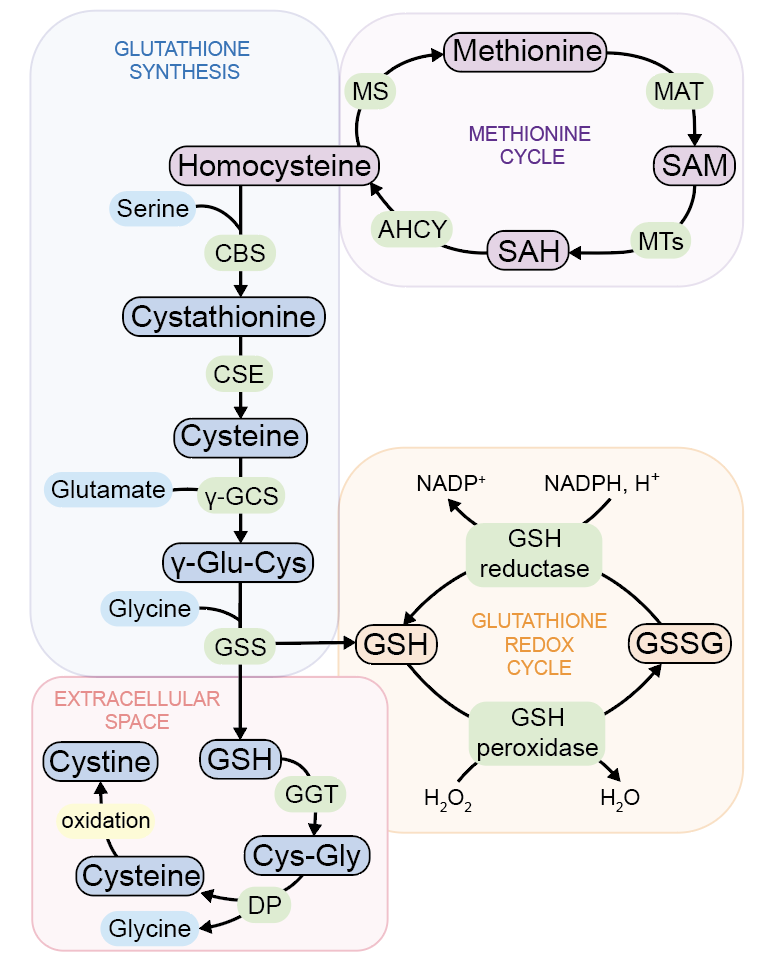
Figure S15.** Metabolites of the methionine cycle, glutathione synthesis and the glutathione redox cycle. Methionine is converted to S-adenosyl-methionine (SAM) by methionine adenyosyltransferase (MAT). SAM is used as a methyl source by methyltransferases (MTs) and S-adenosyl-homocysteine (SAH) is formed as a by-product. SAH is converted by adenosylhomocysteine (AHCY) to homocysteine, which is converted to methionine by methionine synthase (MS) or directed to the glutathione synthesis pathway (Sanderson et al., 2019). Cystathionine β-synthase (CBS) catalyses the addition of homocysteine and serine to generate cystathionine, which is degraded to cysteine by cystathionine γ‐lyase (CSE). γ‐glutamyl cysteine synthetase (γ‐GCS) catalyses the combination of cysteine and glutamate to generate γ-glutamylcysteine (γ-Glu-Cys). Glutathione synthetase (GSS) catalyses the condensation of γ-Glu-Cys and glycine to form glutathione (GSH) (Paul, 2021). GSH peroxidase reduces hydrogen peroxide (H_2_O_2_) and uses GSH as a co-factor, resulting in the formation of oxidised glutathione (GSSG). GSH reductase reduces GSSG to GSH by using NADPH (Lubos et al., 2011). GSH exported into the extracellular space is converted to cysteinylglycine (Cys-Gly) by γ-glutamyltransferase (GGT). Cys-Gly is converted to cysteine and glycine by dipeptidase (DP). In the extracellular space, cysteine is readily oxidised to cystine, which is imported into the cell for GSH synthesis (Banjac et al., 2008).

**S1. Methods**

*S1.1. Vascular reactivity ex vivo*

Naïve male Sprague-Dawley rats (aged ~8-10 weeks) were culled by and obtained from other investigators that did not require the mesenteric arcade for their own studies. Ethics approval was obtained from the Animal Ethics Committee of Monash University (AEC; approval no. 22640) and rats were sourced from the Animal Resources Centre (ARC; WA, Australia). Rats were housed at the Monash Institute of Pharmaceutical Sciences (MIPS) campus of the Monash Animal Research Platform and maintained on a 12 h light/dark cycle at room temperature (18-23ºC) with food (standard laboratory chow) and water provided ad libitum. Rats were anaesthetised by ketamine/xylazine (100/12 mg/kg, i.p.) and euthanised by exsanguination via rapid excision of the heart. The mesenteric arcade was isolated and immediately placed in ice-cold PSS (mmol/L: 120 NaCl, 5 KCl, 1.2 MgSO_4_, 1.2 KH_2_PO_4_, 25 NaHCO_3_, 11.1 D-glucose, 2.5 CaCl_2_) containing 0.01 mmol/L indomethacin. Small mesenteric arteries (second-order branches of the superior mesenteric artery) were isolated, cleared of fat and loose connective tissue, cut into rings 2 mm in length, and mounted on a 4 channel Mulvany-Halpern wire myograph (model 610M; Danish Myo Technology, Aarhus, Denmark). All wire myography experiments were performed using Krebs’ solution with and without EDTA (0.026 mmol/L) at 37°C with organ baths continuously bubbled with carbogen (95% O_2_ and 5% CO_2_). Arteries were allowed to stabilise at zero tension for 30 min prior to normalisation, which was performed by stretching arteries in increments to achieve a final tension equivalent to 70 mmHg. Thirty minutes following normalisation, arteries were maximally contracted by brief exposure to high K^+^ physiological saline solution (KPSS; mmol/L: 25 NaCl, 100 KCl, 1.2 MgSO_4_, 1.2 KH_2_PO_4_, 25 NaHCO_3_, 11.1 D-glucose, and 2.5 CaCl_2_). Then, arteries were rinsed and pre-contracted to 70-80% of their maximal contraction to KPSS using a combination of the thromboxane A_2_ mimetic U46619 (0.1 µmol/L) and the selective α_1_-adrenoceptor agonist phenylephrine (0.5 to 5 µmol/L). Integrity of the endothelium was assessed by exposing the vessel to a single concentration of the endothelium-dependent vasodilator acetylcholine (ACh; 10 µmol/L). Arteries were then washed and precontracted to similar levels (70-80% of KPSS response) using U46619 (0.1 µmol/L) and phenylephrine (0.5 to 5 µmol/L), followed by construction of cumulative concentration-response curves to the NO• donor DEA/NO (0.1 nmol/L to 50 µmol/L), the HNO donor Angeli’s salt (0.1 nmol/L to 50 µmol/L) or sodium nitrite (0.1 nmol/L to 50 µmol/) in the presence or absence the NO• scavenger hydroxocobalamin (HXC; 100 µmol/L, 15 min pre-incubation) or the HNO scavenger L-cysteine (3 mmol/L, 5 min pre-incubation).

**S2. Results**

*S2.1. Impact of EDTA on relaxation responses in mesenteric arteries*

In the presence or absence of EDTA, DEA/NO-mediated relaxation was unaltered by L-cysteine (Figure S14A,B) but was markedly attenuated by HXC (Figure S14A,B; *P*<0.05 vs. control). In the presence of EDTA, L-cysteine caused a significant rightward shift in the concentration-response curve to Angeli’s salt (Figure S14C; *P*<0.05 vs. control). This inhibitory effect of L-cysteine on relaxation to Angeli’s salt was not evident when EDTA was excluded from the Krebs’ buffer (Figure S14D). Angeli’s salt-mediated relaxation was unchanged by HXC in the presence or absence of EDTA (Figure S14C,D). Relaxation responses to sodium nitrite were unaffected by HXC or L-cysteine in the presence or absence of EDTA (Figure S14E,F). Sodium nitrite was less potent as a vasodilator compared to Angeli’s salt in the presence (sodium nitrite: pEC_50_=5.13±0.06; Angeli’s salt: pEC_50_=6.28±0.08, *P*<0.05) or absence (sodium nitrite: pEC_50_=5.33±0.11; Angeli’s salt: pEC_50_=6.37±0.22, *P*<0.05) of EDTA (Figure S14C-F and Table S3).

**S3. Supplementary References**

Banjac, A., Perisic, T., Sato, H., Seiler, A., Bannai, S., Weiss, N., Kölle, P., Tschoep, K., Issels, R. D., Daniel, P. T., Conrad, M., & Bornkamm, G. W. (2008). The cystine/cysteine cycle: a redox cycle regulating susceptibility versus resistance to cell death. *Oncogene*, *27*(11), 1618–1628. https://doi.org/10.1038/sj.onc.1210796

Lubos, E., Loscalzo, J., & Handy, D. E. (2011). Glutathione peroxidase-1 in health and disease: From molecular mechanisms to therapeutic opportunities. *Antioxidants and Redox Signaling*, *15*(7), 1957–1997. https://doi.org/10.1089/ars.2010.3586

Paul, B. D. (2021). Neuroprotective roles of the reverse transsulfuration pathway in alzheimer’s disease. *Frontiers in Aging Neuroscience*, *13*, 1–9. https://doi.org/10.3389/fnagi.2021.659402

Sanderson, S. M., Gao, X., Dai, Z., & Locasale, J. W. (2019). Methionine metabolism in health and cancer: a nexus of diet and precision medicine. *Nature Reviews Cancer*, *19*(11), 625–637. https://doi.org/10.1038/s41568-019-0187-8
